# NTRK Gene Fusions in Solid Tumors and TRK Inhibitors: A Systematic Review of Case Reports and Case Series

**DOI:** 10.3390/jpm12111819

**Published:** 2022-11-02

**Authors:** Giovanni Maria Iannantuono, Silvia Riondino, Stefano Sganga, Roberto Rosenfeld, Simona Guerriero, Manuela Carlucci, Barbara Capotondi, Francesco Torino, Mario Roselli

**Affiliations:** Medical Oncology Unit, Department of Systems Medicine, University of Rome Tor Vergata, Via Montpellier 1, 00133 Rome, Italy

**Keywords:** NTRK gene fusions, TRK inhibitors, agnostic therapy, precision medicine

## Abstract

The approval of larotrectinib and entrectinib for cancer patients harboring an NTRK gene fusion has represented a milestone in the era of “histology-agnostic” drugs. Among the clinical trials that led to the approval of these two drugs, most of the enrolled patients were affected by soft tissue sarcomas, lung, and salivary gland cancer. However, as next-generation sequencing assays are increasingly available in the clinical setting, health care professionals may be able to detect NTRK gene fusions in patients affected by tumor types under or not represented in the clinical trials. To this aim, we systematically reviewed MEDLINE from its inception to 31 August 2022 for case reports and case series on patients with NTRK gene fusion-positive tumors treated with TRK inhibitors. A virtual cohort of 43 patients was created, excluding those enrolled in the above-mentioned clinical trials. Although our results align with those existing in the literature, various cases of central nervous system tumors were registered in our cohort, confirming the benefit of these agents in this subgroup of patients. Large, multi-institutional registries are needed to provide more information about the efficacy of TRK inhibitors in cancer patients affected by tumor types under or not represented in the clinical trials.

## 1. Introduction

### 1.1. Rationale

In the last decade, the advent of precision medicine has revolutionized the treatment landscape of several solid tumors [1]. The rapidly expanding knowledge in genomics, proteomics, and transcriptomics has led to the discovery of new molecular alterations and markers of immune phenotypes shared by multiple tumor types regardless of their site of origin [2]. In this scenario, the availability of pharmacological agents specifically and selectively targeting those alterations and markers has led to the approval of the so-called “histology-agnostic” drugs [3]. This new therapeutic approach has determined a paradigm shift in cancer treatment, paving the way for a new class of biomarker-driven anticancer agents that transcend tumor histologies [4]. Since 2017, the Food and Drug Administration (FDA) has approved six anticancer agents with a “histology-agnostic” indication: two immune checkpoint inhibitors (for both cancers with high tumor mutational burden or mismatch-repair deficiency/microsatellite instability) [5,6,7] and four targeted therapies (for tumors harboring a BRAF V600E mutation or a neurotrophic tyrosine receptor kinase (NTRK) gene fusion) [8,9,10].

The NTRK gene family is composed of three members (NTRK1, NTRK2, and NTRK3) that encode for three tropomyosin receptor kinases (TRK) (TrkA, TrkB, and TrkC, respectively), physiologically involved in the development of the central and peripheral nervous system [11]. The occurrence of NTRK gene rearrangements determines a constitutive activation of TRK receptors, potentially leading to cancer cell transformation [11]. In this framework, several studies have recently demonstrated that these alterations are oncogenic drivers of several tumors [12]. They can be detected with high frequencies (up to 90%) in some rare cancers (secretory breast carcinoma, mammary analogue secretory carcinoma, congenital infantile fibrosarcoma) and with lower frequencies (less than 1%) in a range of common adult patients’ cancers (non-small cell lung, colorectal, thyroid, and salivary gland cancers) [13].

Two oral targeted agents are currently available for unresectable locally advanced or metastatic cancer patients harboring an NTRK gene fusion: larotrectinib [9] and entrectinib [10]. The former was approved in 2018, based on the results of a prespecified combined analysis of three clinical trials published by Drilon et al. [14]. The latter was approved in 2019, after the publication of a pooled analysis of three clinical trials by Doebele et al. [15]. Although the above clinical trials enrolled patients with NTRK gene fusion-positive tumors regardless of their site of origin, most were affected by soft tissue sarcomas, lung, and salivary gland cancer [14,15]. In contrast, some tumor types were under or not represented, such as gastrointestinal (except for colorectal cancer), genitourinary, and gynecological malignancies [14,15]. However, as genomic medicine is constantly evolving, next-generation sequencing (NGS) assays for comprehensive genomic profiling have become increasingly available in clinical and research settings [16]. Therefore, health care professionals may have the chance to detect an NTRK gene fusion even in patients with tumor histologies under or not represented in clinical trials [14,15], thus facing the opportunity to provide patients with TRK inhibitors despite a lack of data.

### 1.2. Objective

To this aim, we evaluated the benefit of TRK inhibitors in a virtual cohort of patients affected by NTRK gene fusion-positive solid tumors derived from case reports (CR) and case series (CS) available in the literature. Although CR and CS are characterized by a high risk of biases due to their nature, they have consistently advanced medical knowledge on rare conditions, providing helpful information for clinical practice. To our knowledge, CR and CS on cancer patients harboring an NTRK gene fusion and treated with TRK inhibitors have never been systematically reviewed.

## 2. Methods

### 2.1. Protocol and Registration

The literature search was conducted according to the *Preferred Reporting Items for Systematic Reviews and Meta-analyses* (PRISMA) guidelines for systematic reviews [17] (Supplementary Material S1). The protocol was designed a priori by all the authors and was registered on the Open Science Framework website (https://osf.io/ngz6s/ [accessed on 1 October 2022]).

We included CR and CS on pediatric and adult patients affected by locally advanced or metastatic NTRK gene fusion-positive solid tumors treated with larotrectinib or entrectinib. Only publications in English or European languages were considered. Furthermore, CS were deemed eligible only if single-case descriptions were provided. Letters to the editor or other types of publications reporting CR or CS were also considered if they satisfied all the previous criteria. On the contrary, CR and CS on NTRK gene fusion-positive solid tumor patients treated with TRK inhibitors not yet approved by the FDA were excluded. In addition, eligible publications reporting on patients enrolled in the clinical trials that led to the approval of larotrectinib and entrectinib were excluded, as well [14,15]. Indeed, this systematic review aimed to create a virtual cohort of patients different from the populations enrolled in the clinical trials. Finally, patients who received TRK inhibitors through an early access program or were enrolled in observational or non-interventional clinical trials (e.g., post-marketing safety surveillance studies) were considered eligible.

### 2.2. Search

The electronic PubMed database was searched from inception to 31 August 2022 to identify all relevant publications. No research filters were used. The search strategy was decided on after a discussion among the authors. It was composed of four different syntaxes that were consecutively evaluated: (i) larotrectinib AND (case report OR case series); (ii) entrectinib AND (case report OR case series); (iii) larotrectinib AND NTRK; (iv) entrectinib AND NTRK. The results were uploaded to a reference management software (Zotero), and duplicates were later removed.

Two groups of three authors (G.M.I., M.C., S.G. and S.R., S.G., B.C.) independently scrutinized the available results following a two-stage study selection process. Firstly, all titles and abstracts were screened for potential relevance. Secondly, full texts of potentially appropriate results were retrieved and further assessed for eligibility. An agreement of the three authors of each group was required for exclusion at both stages. At the end of the selection process, the two groups discussed the list of publications to include in the review. In parallel, all the references of eligible studies were also hand-searched for other potential publications. In case of disagreements on the study selection, a consultation with two additional authors (M.R. and F.T.) was required. Finally, the two groups of authors achieved a complete consensus on the included papers and reported the results in the PRISMA flow diagram [17].

### 2.3. Data Charting Process

Three authors (G.M.I., S.R., F.T.) created a data charting template using Microsoft Excel software. As in the study selection process, the two groups of authors charted the data independently, discussing the results in an interactive process. Once a CS was analyzed, the single-case data were extracted individually. Disagreements in the charting process required consultation with two additional authors (M.R. and F.T.) and were resolved by consensus.

The variables extracted were: first author, journal of publication, year of publication, type of evidence (CR or CS), age and sex of the patient, primary tumor type, histological subtype, site of metastases (before being treated with NTRK inhibitors), NTRK gene fusion diagnostic modality, type of NTRK gene fusion, previous treatments, type of TRK inhibitor received, best radiological response (according to Response Evaluation Criteria in Solid Tumors (RECIST)), and outcomes.

Variables charted from eligible publications were described using numbers and proportions for categorical variables while mean, standard deviation, median, and interquartile range were used for continuous variables. The descriptive analyses were performed using R Studio (version 1.4.1106) software. Quantitative analyses were performed considering the overall population composed of both adult and pediatric patients, as reported in the clinical trials that led to the approval of larotrectinib and entrectinib. No inferential or predictive statistics analyses were performed.

### 2.4. Risk of Bias Assessment

The risk of bias assessment of included publications was performed using the methodological tool proposed by Murad et al. [18]. The two groups of authors responsible for the “selection process” assigned a binary response (yes = 1 and no = 0) to every question required by the tool. Subsequently, an aggregated score was formulated for each included CR or CS. In case of disagreements, a consultation with two additional authors (M.R. and F.T.) was required.

## 3. Results

### 3.1. Study Characteristics

**Figure 1:**
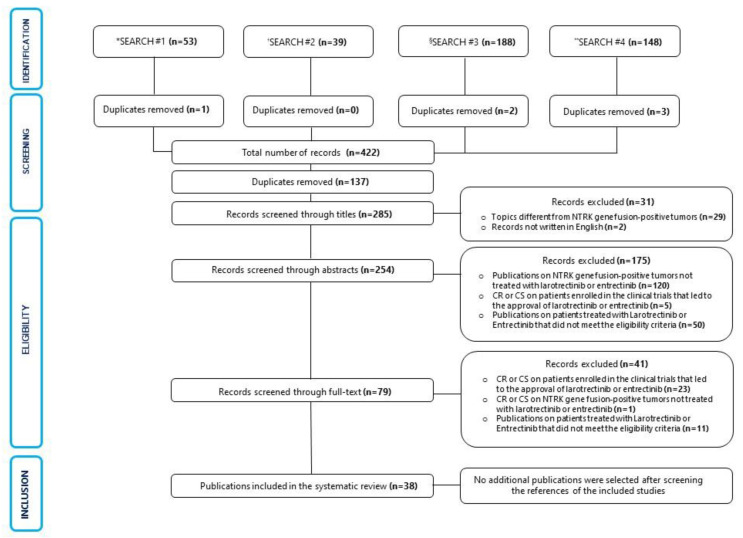
Flowchart of the results of literature search and selection process of included publications. * Larotrectinib AND (case report OR case series). ^†^ Entrectinib AND (case report OR case series). ^§^ Larotrectinib AND NTRK. ** Entrectinib AND NTRK. Abbreviations: Case report (CR), Case series (CS).

The results of the literature search and the study selection process are displayed through a PRISMA diagram in Figure 1.

**Figure 2:**
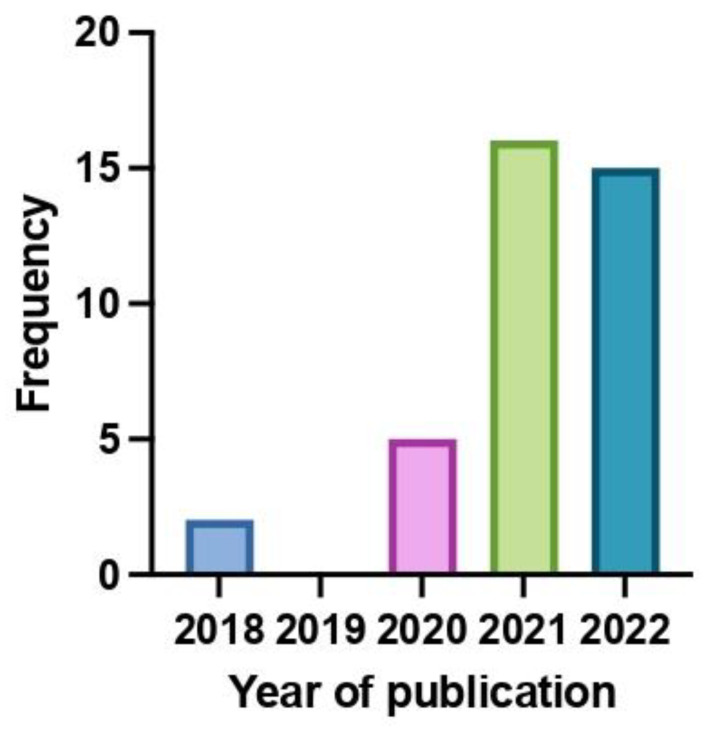
Distribution of included publications based on the year of publications.

A total of 38 publications were included in this systematic review: 32 CR and 6 CS. They were published between 2018 and 2022 (Figure 2). Particularly, 21 CR and 2 CS reported about adult patients, while 11 CR and 4 CS were on pediatric patients. All the publications were written in English. Among the ineligible publications, 28 CR/CS were excluded because they reported data about patients enrolled in the clinical trials that led to the approval of larotrectinib and entrectinib. Furthermore, 1 CR was excluded because the patient was affected by a hematologic tumor. The results of the included single sources of evidence are described in Table 1.

### 3.2. Synthesis of Results

Data extracted from the included CR and CS allowed us to create a virtual cohort of 43 patients affected by NTRK gene fusion-positive tumors treated with TRK inhibitors, including 25 adult and 18 pediatric patients (Table 2).

The median age in the overall population was 37 years (range: <1–81 years). The median age for adult patients was 56 years (range: 26–81), while for pediatric patients, it was 4 years (range: <1–14). Furthermore, 19 (44.2%) patients were male (11 adult and 8 pediatric patients) and 21 (48.8%) female (14 adult and 7 pediatric patients). Sex was not reported in 3 cases (7%). In the overall population, the frequencies of tumor types harboring an NTRK gene fusion were soft tissue sarcoma (30.2%), central nervous system (CNS) tumor (27.9%), thyroid tumor (14%), salivary gland tumor (9.3%), lung cancer (4.8%), cervical cancer (2.3%), breast cancer (2.3%), colon cancer (2.3%), ovarian cancer (2.3%), pancreatic tumor (2.3%), and thymoma (2.3%). Among adult patients, the most frequent tumor types were thyroid tumors (24%), salivary gland tumors (16%), and CNS tumors (16%) while for pediatric patients, they were soft tissue sarcoma (55.6%) and CNS tumors (44.4%). Furthermore, the majority of patients (79.1%) were treated with TRK inhibitors for a metastatic disease. The metastatic sites in the overall population were lungs (38.2%), lymph nodes (38.2%), bone (26.5%), liver (20.6%), pleura (14.7%), soft tissues (14.7%), brain (8.8%), adrenal gland (8.8%), peritoneum (8.8%), kidney (5.9%), leptomeninges (5.9%), pancreas (5.9%), mediastinum (5.9%), and ovary (2.9%). In addition, 38.2% of patients had loco-regional recurrence.

In the overall population, the detected NTRK gene rearrangements involved NTRK1, NTRK2, and NTRK3 in 25.6%, 16.3%, and 51.2% of cases, respectively. However, in 7% of cases, the authors did not report the specific NTRK gene involved. The most common NTRK fusion partners were ETV6 (37.2%), TMP3 (7%), and EML4 (7%). NTRK gene fusions were detected in the primitive tumor or distant metastases’ specimens in 69.7% and 16.3% of cases, respectively. Among the methodologies for NTRK gene fusions’ detection, immunohistochemistry (IHC), fluorescent in situ hybridization (FISH), DNA- or RNA-based NGS assays, and reverse transcription–polymerase chain reaction (RT-PCR) were used in 27.9%, 18.6%, 83.7%, and 0% of cases, respectively. No information was reported in 16.6% of cases on the methodologies used for NTRK gene fusion’s detection. Before receiving an TRK inhibitor, 90.7% of patients were treated with other treatments, particularly, surgery (67.4%), chemotherapy (39.5%), targeted therapies (20.9%), immune checkpoint inhibitors (4.7%), radiotherapy (27.9%), radioactive iodine therapy (9.3%), and chemoradiotherapy (14%).

In terms of treatment, larotrectinib and entrectinib were administered in 81.4% and 16.3% of patients, respectively. Furthermore, in one case (2.3%), the patient was treated with both drugs. TRK inhibitors were used as first- and second-line therapy in 34.9% of cases. In addition, in 16.3% of cases, TRK inhibitors were used as third line and, in 6.9% of cases, they were used in the subsequent lines. The best radiological response to TRK inhibitors was partial response (74.5%), while a complete response was achieved in 20.9% of cases. In contrast, only 2.3% of cases had stable or progressive disease, respectively. Finally, at the time of publication, 72.1% patients were alive with disease, 11.6% died due to progressive disease, and no information was reported for 16.3% of cases.

### 3.3. Quality Assessment

All the included publications were evaluated with a tool proposed by Murad et al. [18]. The aggregated scores assigned to every CR and CS included in the study are available in Supplementary Material S2.

## 4. Discussion

### 4.1. Summary of Evidence

In recent years, the advances in molecular diagnosis have led to a significant change in how cancer patients are treated, shifting from a “one size fits all” therapeutic paradigm toward a “precision medicine” approach with the development of new agents targeting specific genomic abnormalities [4,5,6,7,8,9,10,11,12,13,14,15,16,17,18,19,20,21,22,23,24,25,26,27,28,29,30,31,32,33,34,35,36,37,38,39,40,41,42,43,44,45,46,47,48,49,50,51,52,53,54,55,56,57,58]. This revolution has been witnessed by the progressive discovery of an increasing number of actionable molecular alterations, gaining the chance of improving cancer patients’ survival with biomarker-driven drugs [1]. Nowadays, several “agnostic therapies” have been approved for patients harboring specific genomic alterations, based on the possibility of administering targeted therapies across different tumor histologies and regardless of the tumor site of origin [59].

**Figure 3:**
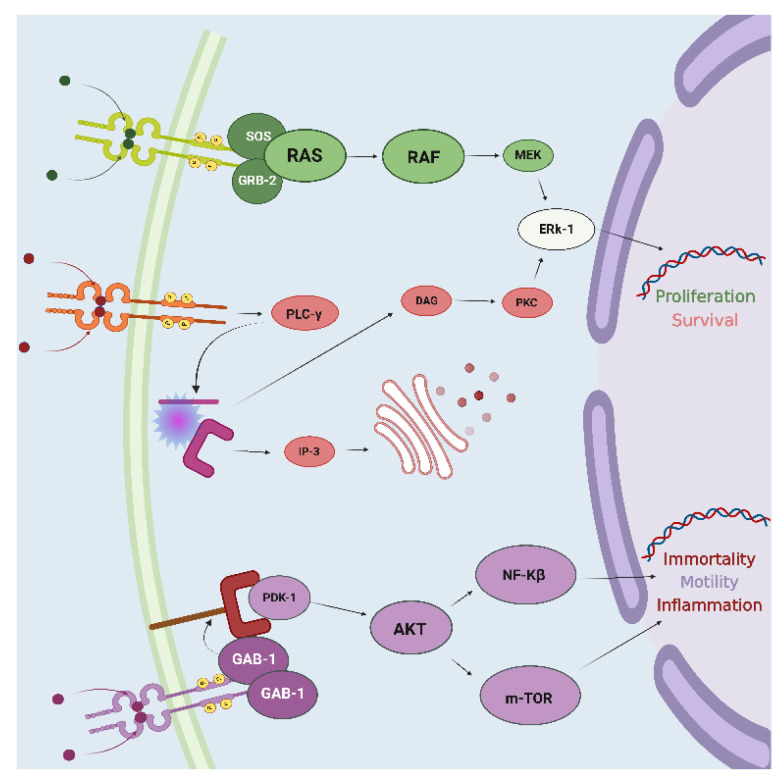
TRK receptors’ intracellular signaling pathway. The interaction between the receptor and the ligand activates crosstalk between multiple intracellular molecular cascades. Abbreviations: protein kinase B (AKT); Diacylglycerol (DAG); Extracellular signal-regulated kinase (ERk-1); GRB2-Associated Binding Protein-1 (GAB-1); Growth Factor Receptor-Bound Protein-2 (GRB-2); Inositol trisphosphate (IP-3); Mitogen-activated protein kinase (MEK); Mammalian Target of Rapamycin (m-TOR); Nuclear Factor Kinase-β (NF-Kβ); 3-Phosphoinositide-Dependent Protein Kinase-1 (PDK-1); Protein Kinase C (PKC); Phospholipase C-γ (PLC-γ); Rapidly Accelerated Fibrosarcoma (RAF); Rat Sarcoma virus (RAS); Son of Sevenless (SOS). Created with BioRender.com.

In recent years, NTRK gene fusion has represented one of the most groundbreaking discoveries among the biomarkers targeted by agnostic therapies. The family of NTRK genes (NTRK1, NTRK2, NTRK3) encodes for TrkA, TrkB, and TrkC receptors, respectively [11]. They are composed of an intracellular domain, a transmembrane region, and an extracellular domain for ligand binding [60]. Beyond their physiological involvement in the nervous system’s development, the constitutive activation of Trk receptors mediated by the occurrence of NTRK gene fusions leads to the uncontrolled growth of cancer cells [11]. Particularly, the interaction between TRK receptors and their ligands triggers the activation of signal transduction pathways implicated in tumorigenesis, including Ras/Mitogen activated protein kinase (MAPK), the phosphatidylinositol-3-kinase (PI3K)/Akt, and the mammalian target of rapamycin (mTOR) pathways (Figure 3) [61].

In 2013, Vaishnavi et al. described, for the first time, an NTRK1 gene rearrangement in a cohort of patients affected by non-small cell lung cancer [62]. Since then, several studies aiming to describe the genomic landscape and the prevalence of NTRK gene fusions in solid tumors have been published. In 2019, Rosen et al. reported the analysis of genomic and clinical data about NTRK gene-positive tumors identified among more than 26,000 prospectively sequenced patients. Seventy-six cases (0.28%) with confirmed NTRK fusions were identified, mainly represented by salivary gland cancer, soft tissue sarcomas, and thyroid cancers [63]. In 2020, Forsythe et al. reported the results of a systematic review and meta-analysis aiming at describing the NTRK gene fusion incidence among available studies published from 1987 to 2020. The authors showed that rare tumors, such as secretory breast cancer, infantile fibrosarcoma, secretory salivary gland cancer, papillary thyroid carcinoma (pediatric), and congenital mesoblastic nephroma, were characterized by the highest NTRK gene fusion frequencies (from 10 to 92.8%) [64]. In 2021, Westphalen et al. reported the results of a retrospective study which aimed to evaluate the genomic landscape and prevalence of NTRK gene fusions in a large real-world database of comprehensive genomic profiling data (FoundationCORE). Among more than 295,000 analyzed cancer cases, salivary gland cancers (2.43%), soft tissue sarcomas (1.27%), and thyroid cancers (1.25%) were the most common tumor types harboring NTRK gene fusions [65].

From a diagnostic point of view, several assays have been developed to accurately identify patients harboring an NTRK gene fusion, including IHC, FISH, RT-PCR, and DNA- or RNA-based NGS. However, the advantages and disadvantages of each diagnostic modality must be weighed when evaluating the tissue specimen required for NTRK gene fusions analysis [66]. To this aim, the European Society of Medical Oncology Translational Research and Precision Medicine Working Group published recommendations for a rational approach for detecting NTRK gene fusions based on the prevalence of these alterations among different tumor histotypes [67]. In tumors with highly frequent NTRK fusions, the best methodologies to use as confirmatory tests are FISH, RT-PCR, or targeted RNA NGS assays. Differently, in tumors where NTRK fusions are recognized not to be highly prevalent, NGS targeted panels (DNA- or RNA-based) are the recommended tests of choice. In case of an NTRK gene fusion detection by the use of NGS-based assays, a further confirmatory test with IHC is recommended [67]. Alternatively, if a sequencing platform is not available, IHC may be used as a screening tool, followed by an NGS targeted panel, in case of a positive result [67].

Nowadays, two targeted agents are available for patients harboring NTRK gene fusions: larotrectinib and entrectinib. The former was granted accelerated approval by the FDA in 2018 [9] after Drilon et al. published a prespecified combined analysis of three clinical trials evaluating the activity of larotrectinib in patients with locally advanced or metastatic NTRK fusion-positive solid tumors. The studies involved in the analysis were a phase 1 trial on adults (LOXO-TRK-14001), a phase 1–2 trial on children (SCOUT), and a phase 2 “basket” trial involving adolescents and adults (NAVIGATE). Among the 55 patients enrolled, the overall response rate was 75% (95% CI, 61–85%) after a median follow-up of 9.4 months [14]. After two years, Hong et al. reported the results of an expanded pooled efficacy analysis on 159 patients enrolled across the same 3 clinical trials. Although only 153 patients were evaluable for response, the objective response rate was 79% (95% CI, 72–85%) after a median follow-up of 11.1 months. In total, 24 (16%) patients achieved a complete response, and the median progression-free survival was 28.3 months (95% CI, 22.1—not reached) [68]. Recently, Drilon et al. updated the previous results by publishing the efficacy analysis on 244 patients. The objective response rate was 69% (95% CI, 63–75%), with a complete response rate of 26%. The median progression-free survival was 29.4 months (95% CI, 19.3–34.3 months) after a median follow-up of 29.3 months [69]. In contrast, entrectinib was granted accelerated approval by the FDA in 2019 [10] after Doebele et al. published a pooled analysis of three clinical trials that investigated the activity of entrectinib in locally advanced or metastatic cancer patients harboring an NTRK gene fusion. The studies involved in the analysis were two phase I trials (ALKA-372–001 and STARTRK-1) and one phase II trial (STARTRK-2). The objective response rate was 57% (95% CI, 43.2–70.8%) among the enrolled 54 patients, after a median follow up of 12.9 months. The complete response rate was 7% and the median duration of response was 10 months (95% CI, 7.1–NE) [15].

Overall, the population included in the above-mentioned clinical trials was composed of patients affected by different tumor histologies harboring an NTRK gene fusion. The most frequent tumor histotypes were soft tissue sarcomas, lung, salivary gland, and thyroid cancer. In contrast, gastrointestinal (except for colorectal cancer), genito-urinary, and gynecological malignancies were under or not-represented [14,15]. However, since the rapid evolution of precision medicine has determined a reduction in the cost of molecular profiling, NGS assays have become increasingly available in clinical practice. Accordingly, health care professionals are more likely to face the detection of NTRK gene fusions in patients affected by tumor histologies under or not represented in the clinical trial, with the consequent lack of data on the efficacy of TRK inhibitors. Therefore, we systematically reviewed the available literature for CS and CR on NTRK gene fusion-positive tumors treated with TRK inhibitors.

The publication of CR and CS has often played an essential role in advancing medical knowledge on rare conditions [18,19,20,21,22,23,24,25,26,27,28,29,30,31,32,33,34,35,36,37,38,39,40,41,42,43,44,45,46,47,48,49,50,51,52,53,54,55,56,57,58,59,60,61,62,63,64,65,66,67,68,69,70]. In this context, the creation of a virtual cohort of patients from CR and CS on NTRK gene fusion-positive solid tumors treated with TRK inhibitors has a double value. On the one hand, it provides health care professionals with a single source of evidence that is easy to access and summarizes data derived from multiple clinical experiences. On the other hand, the choice to exclude both CR and CS on patients enrolled in the clinical trials lends this virtual cohort of patients the opportunity to be compared with populations enrolled in the clinical trials [14,15]. Nevertheless, since the intrinsic bias of both CR and CS is related to their nature and the inclination to publish more reports of positive rather than negative responses to treatments, it is essential to be cautious in the extrapolation of emerging data to clinical practice [18].

The data obtained from this patients’ cohort appear in line with those available in the current literature. Indeed, patients were affected by the most frequent tumor types as those reported in the clinical trials that led to the approval of larotrectinib and entrectinib [14,15], such as soft tissue sarcoma, thyroid, and salivary gland tumors (Table 3). In our opinion, a reasonable explanation for these similar results relies on the different frequencies of NTRK gene fusions among different tumor histotypes, as reported in the above-mentioned multiple retrospective studies [63,64,65]. Although the NTRK gene fusions are rare genomic abnormalities, they can be detected with high frequencies in some rare cancers (secretory breast carcinoma, mammary analogue secretory carcinoma, congenital infantile fibrosarcoma) and with lower frequencies in a range of common adult patients’ cancers [13].

In our cohort, we found a higher prevalence of patients affected by primary CNS tumors (27.9%) compared to those reported in the abovementioned clinical trials’ combined analyses. Nowadays, these patients lack effective therapies [71] and, thus, our results confirm the indication of the current guidelines that encourage performing molecular testing for NTRK gene fusion in CNS tumors [72]. However, it is important to cautiously interpret the extremely positive outcomes reported by the patients included in our cohort due to the “publication bias” related to CR and CS. In this direction, a recently published post-hoc analysis of two clinical trials that led to the approval of larotrectinib showed an objective response rate of 30% (95% CI, 16–49) with a 24-week disease control rate of 73% (95% CI: 54–87) for primary CNS tumors [73]. Nevertheless, considering the reported benefit of administering TRK inhibitors in this subgroup of patients and the data available in the literature, the detection of an NTRK gene fusion in CNS tumors may represent a “game-changer” in treating those malignancies.

Finally, our results confirm how TRK inhibitors represent a significant therapeutic strategy for metastatic cancer patients harboring an NTRK gene fusion, and, thus, all the cancer patients harboring this molecular alteration should be evaluated for specific inhibitors [74]. Although these drugs are generally administered as a single-agent treatment in the metastatic setting, we found a cervical cancer patient treated with the combination of chemotherapy plus larotrectinib [30] and two pediatric patients treated with “adjuvant” maintenance larotrectinib after definitive surgical resection of a kidney sarcoma and anaplastic astrocytoma [38]. These reports are interesting because they focus on new potential strategies of TRK inhibitors’ administration in terms of clinical setting (adjuvant vs. metastatic disease) and combinations (single agent vs. combined treatment). In our opinion, considering the importance of maximizing the benefit of these drugs, we believe that these strategies should be further assessed in the near future. In parallel, it is essential to provide guidelines to help healthcare professionals determine the best time to administer these anticancer agents. Recently, a Belgian expert consensus for the tumor-agnostic treatment of NTRK gene fusion-driven solid tumors with larotrectinib has been published [75]. The authors distinguished three categories of patients affected by NTRK gene fusion-driven solid tumors: (i) those affected by advanced solid tumors with non-satisfactory standard-of-care (SoC) therapies, (ii) those with advanced solid tumors with satisfactory SoC therapies, and (iii) those affected by locally advanced tumors [75]. For the first group, it was suggested to consider the use of larotrectinib as a first-line treatment, considering the high unmet medical need for these patients. Concerning the second group, the authors suggested larotrectinib as a consideration for second or later treatment line (after failure of SoC). For the last group, larotrectinib should be considered as a neoadjuvant therapy [75].

### 4.2. Future Perspectives

We believe that the creation of prospective, international patients’ registries would represent an essential tool to acquire “real-word” data on the efficacy of TRK inhibitors among NTRK gene fusion positive tumors, including histotypes under or not represented in previous clinical trials. Currently, two ongoing studies aim to collect data prospectively in these patients: the REALTRK registry [76] and the TRacKING registry [77]. The former aims to analyze the treatment reality and outcomes of NTRK gene fusion-positive patients treated with TRK inhibitors until at least 36 months after their inclusion in the study [76]. The latter aims to study the real-life management of patients with rare actionable fusions, including those harboring an NTRK gene fusion [77].

### 4.3. Limitations

The present systematic review has some limitations. Firstly, a limited number of CR and CS lacked relevant clinical data and were at high risk of several biases. Secondly, we did not include CS reporting on aggregate patients’ data instead of individual data. Unfortunately, these limitations are common in studies that evaluate CR and CS [78,79], but they were considered before designing the protocol. In addition, the search strategy was designed to be extensive, and both the data extraction and selection were performed with a “two-stage process” to minimize bias.

## 5. Conclusions

The results of our systematic review confirmed the efficacy of TRK inhibitors in cancer patients harboring an NTRK gene fusion. Although the patients of our virtual cohort were mainly affected by the most frequently diagnosed tumor histologies in patients enrolled in the available clinical trials on TRK inhibitors, we reported a higher prevalence of CNS tumors, confirming the benefit of these agents even in this subgroup of patients. Large, multi-institutional registries are needed to provide more information about the efficacy of TRK inhibitors in cancer patients affected by under or not represented histologies included in the clinical trial that led to the approval of entrectinib and larotrectinib.

## Figures and Tables

**Table 1:** Variables extracted from eligible publications.

Publication			Patients’ Characteristics						Treatments and Outcomes			
First Author	Journal of Publication (Year)	Type of Evidence	Age (Sex)	Tumor Type	Histology	Sites of Metastases	NTRK Gene Fusion	Diagnosis	Previous Treatments	NTRK Inhibitor (Line of Therapy)	Best Radiological Response (Duration of Response)	Outcome
Landman et al. [19]	Clin Breast Cancer (2018)	CR	37 (F)	BC	Secretory breast carcinoma	Bone, lung, lymph nodes, peritoneum, pleura	ETV6-NTRK3	NGS	Surg-RT	Laro (1)	PR (6 *)	AwD
Ziegler et al. [20]	Br J Cancer (2018)	CR	3 (F)	CNS	High-grade glioma	Loco-regional recurrence	ETV6–NTRK3	NGS	Surg-ChT-RT	Laro (2)	PR (9 *)	AwD
Wong et al. [21]	Pathology (2020)	CS	65 (F)	STS	Fibrosarcoma	Adrenal gland, kidney, liver, pancreas	ETV6-NTRK3	FISH-IHC-NGS	Surg-RT	Entre (1)	PR (1 *)	AwD
Hochmair et al. [22]	Clin lung cancer (2020)	CR	30 (F)	LC	Adenocarcinoma	Bone	TPM3-NTRK1	IHC-NGS	Cht	Laro (2)	PR (4 *)	AwD
Alharbi et al. [23]	JCO Precis Oncol (2020)	CR	2 (F)	CNS	High-grade glioma	Loco-regional recurrence	ETV6-NTRK3	NGS	Surg	Laro (1)	PR (6 *)	AwD
Mayr et al. [24]	J Pers Med (2020)	CS	9 (NA)	STS	Gliosarcoma	Bone, leptomeninges	EML4-NTRK3	NGS	Surg-ChT-RT	Entre (3)	PR (5)	DoD
Walter et al. [25]	Pediatr Blood Cancer (2020)	CR	6 (NA)	CNS	Low-Grade Glioma	No distant metastases	NACC2-NTRK	NA	ChT-TargT	Laro (5)	PR (NA)	NA
Salame et al. [26]	Cureus (2021)	CR	50 (M)	Thymus	Thymoma	Pleura	EIF4B-NTRK3	NGS	ChT	Entre (2)	PR (10 *)	AwD
Zhang et al. [27]	BMC Pulm Med (2021)	CR	60 (F)	LC	Adenocarcinoma	Lung, pleura	NCOR2-NTRK1	IHC- NGS	Surg-ICI	Laro (3)	PR (15 *)	AwD
Gupta et al. [28]	J Natl Compr Canc Netw (2021)	CR	81 (M)	PC	Pancreatic acinar cell carcinoma	Liver, lymph nodes	SEL1L-NTRK1	NGS	Surg-Cht	Laro (2)	PR (13 *)	AwD
Percy et al. [29]	Clin Case Rep (2021)	CR	30 (M)	STS	Spindle cell sarcoma	No distant metastases	SPECC1L-NTRK	IHC-NGS	None	Laro (Neoadj)	PR (8 ^#^)	AwD
Munkhdelger et al. [30]	Int J Surg Pathol (2021)	CR	72 (F)	CC	Basaloid squamous cell carcinoma	Lung	DLG2-NTRK2	NGS	Surg	Laro (1)	PR (NA)	NA
Pircher et al. [31]	Medicine (Baltimore) (2021)	CR	63 (M)	SG	Carcinoma ex pleomorphic adenoma	Lung, lymph nodes	ZCCHC7-NTRK2	NGS	Surg-RT	Laro (1)	SD	AwD
Pitoia et al. [32]	Clin Case Report (2021)	CR	56 (F)	TT	Papillary	Adrenal gland, bone, brain, liver, lymph nodes, lung, pleura, soft tissue	ETV6-NTRK3	NGS	RAI-TargT	Laro (3)	CR (11 *)	AwD
Shepherd et al. [33]	Oncologist (2021)	CR	26 (M)	CNS	Glioblastoma	Loco-regional recurrence	KANK1-NTRK2	NGS-FISH	CT/RT	Laro-Entre (2)	PR (3.5 ^§^)	DoD
Wagner et al. [34]	Diagn Pathol (2021)	CR	38 (M)	SG	Mammary analogue secretory carcinoma	Bone, lungs	ETV6-NTRK3	IHC-FISH-NGS	Surg-Cht -CT/RT	Laro (1)	PR (8 *)	AwD
Boyer et al. [35]	Neuro Oncol (2021)	CR	53 (M)	CNS	High-grade glioma	Loco-regional recurrence	STRN1-NTRK2	NGS	Surg-CT/RT	Laro (2)	CR (11 *)	AwD
Corral Sánchez et al. [36]	Pediatr Hematol Oncol (2021)	CR	<1 (F)	STS	Infantile fibrosarcoma	No distant metastases	ETV6-NTRK3	FISH	None	Laro (1)	CR (14 *)	AwD
Goh et al. [37]	J Oncol Pharm Pract (2021)	CR	14 (M)	STS	Non-rhabdomyosarcoma soft tissue sarcoma	Soft tissues	DCTN1–NTRK1	IHC-NGS	ChT-RT-Surg	Laro (2)	PR (6)	DoD
Carter-Febres et al. [38]	J Pediatr Hematol Oncol (2021)	CS	2 (F)	STS	Undifferentiated embryonal sarcoma	No distant metastases	ETV6-NTRK3	NGS	ChT-Surg	Laro (Adj)	CR (12 *)	AwD
			3 (M)	CNS	High-grade glioma	No distant metastases	NACC2-NTRK2	NGS	Surg-CT/RT	Laro (Adj)	CR (15 *)	AwD
Slomovic et al. [39]	Pediatr Blood Cancer (2021)	CR	<1 (M)	STS	Infantile fibrosarcoma	No distant metastases	ETV6-NTRK	NA	ChT	Laro (2)	PR (14 *)	AwD
Waters et al. [40]	Pediatr Blood Cancer (2021)	CR	2 (M)	CNS	Glioma	Loco-regional recurrence	EML4-NTRK3	NA	Surg-ChT	Laro (2)	PR (12 *)	AwD
Mangum et al. [41]	JCO Precis Oncol (2021)	CR	6 (M)	CNS	Ependymoma	Loco-regional recurrence, leptomeninges	KANK1-NTRK2	NGS	Surg-RT	Laro (1)	PR (10 *)	AwD
Endo et al. [42]	Mol Clin Oncol (2022)	CR	56 (F)	OC	High-Grade Serous Carcinoma	Lymph nodes, peritoneum, pleura, liver	TPM3-NTRK1	NGS	ChT-Surg-TargT	Entre (6)	PD	DoD
Ernst et al. [43]	Curr Oncol (2022)	CR	59 (M)	SG	Mammary analogue secretory carcinoma	Loco-regional recurrence, lung	ETV6-NTRK3	FISH-NGS	Surg	Entre (1)	PR (49 *)	AwD
Recine et al. [44]	Front Oncol (2022)	CR	14 (M)	STS	Dermatofibrosarcoma	Bone, kidney, liver, lung, soft tissue	TPM4-NTRK1	NGS	Surg-RT-TargT	Laro (2)	PR (23 *)	AwD
Bill et al. [45]	Cancer Rep (Hoboken) (2022)	CR	56 (F)	SG	Mammary analogue secretory carcinoma	Lymph nodes	ETV6-NTRK3	IHC-NGS	Surg-CT/RT	Laro (2)	CR (13 *)	AwD
Bargas et al. [46]	Eur J Endocrinol (2022)	CR	50 (F)	TT	Papillary	Lung, ovary, mediastinum, lymph node	SQSTM1-NTRK1	NGS-FISH	Surg-RAI-TargT	Laro (3)	PR (18 *)	AwD
Kasi et al. [47]	Cureus (2022)	CR	43 (F)	CoC	Adenocarcinoma	Lymph nodes, peritoneum	TPR-NTRK1	NGS-IHC	Surg-ICI	Laro (2)	PR (3 ^†^)	AwD
Saliba et al. [48]	Head Neck Pathol (2022)	CR	49 (M)	TT	Secretory carcinoma	Loco-regional recurrence, lymph nodes, lung	ETV6-NTRK3	NGS	Surg	Laro (1)	PR (18)	DoD
Lapeña et al. [49]	European J Pediatr Surg Rep (2022)	CS	<1 (F)	STS	Infantile fibrosarcoma	No distant metastases	ETV6-NTRK3	NA	None	Laro (1)	CR (14 *)	AwD
			<1 (M)	STS	Infantile fibrosarcoma	No distant metastases	ETV6-NTRK3	NA	None	Laro (1)	CR (6 *)	AwD
Groussin et al. [50]	Thyroid (2022)	CS	65 (F)	TT	Papillary	Bone, liver, lymph nodes, lung	EML4-NTRK3	NGS	RAI-TargT	Laro (3)	PR (NA)	NA
			48 (F)	TT	Papillary	Lymph nodes, lung	ETV6-NTRK3	NGS	RAI-TargT	Laro (3)	PR (NA)	NA
			70 (F)	TT	Oxyphilic cell papillary	Brain, bone, lymph nodes, liver, lung, pancreas, soft Tissue	TPM3-NTRK1	NGS	Surg	Laro (1)	PR (NA)	NA
Grogan et al. [51]	Neurooncol Adv (2022)	CR	67 (M)	CNS	Glioblastoma	Loco-regional recurrence	BCR-NTRK2	NGS	Surg-RT	Entre (1)	PR (15)	NA
Kobayashi et al. [52]	Genes Chromosomes Cancer (2022)	CR	57 (M)	STS	Malignant peripheral nerve sheath tumors	Lymph nodes, lung	SNRNP70-NTRK3	FISH-IHC-NGS	Surg-RT-ChT- TargT	Entre (4)	PR (11)	NA
König et al. [53]	Pharmacology (2022)	CR	80 (F)	CNS	High-grade glioma	No distant metastases	ARHGEF7-NTRK3	NGS	RT	Laro (1)	PR (4.5)	AwD
Olsen et al. [54]	J Pediatr Hematol Oncol (2022)	CR	6 (F)	STS	High-grade spindle cell sarcoma	Bone	NTRK3 gene rearrangement	IHC-FISH	ChT-RT	Laro (3)	PR (22)	AwD
Mançano et al. [55]	Pathobiology (2022)	CR	<1 (M)	STS	Gliosarcoma	Loco-regional recurrence	TPR-NTRK1	FISH-IHC-NGS	Surg-ChT	Laro (2)	PR (8 *)	AwD
Di Ruscio et al. [56]	Diagnostics (2022)	CS	1 (NA)	CNS	High-grade glioma	Loco-regional recurrence	ETV6-NTRK3	NGS	Surg-ChT-TargT	Laro (2)	CR (24 *)	AwD
			1 (F)	CNS	High-grade glioma	Loco-regional recurrence	MEF2D-NTRK1	NGS	Surg-ChT	Laro (2)	PR (4 *)	AwD

* Response was ongoing at the time of publication. ^#^ After 8 months of larotrectinib the patient underwent radical surgery. ^§^ The duration of response is related to larotrectinib. ^†^ After 3 months of larotrectinib, the patient underwent radical surgery and, subsequently, the treatment was continued to complete a total of 6 months of peri-operative therapy. Abbreviations: Adjuvant (Adj); Alive with Disease (AwD); Breast Cancer (BC); Case Report (CR); Case Series (CS); Central Nervous System (CNS); Cervical Cancer (CC); Chemotherapy (ChT); Chemoradiotherapy (CT/RT); Colon Cancer (CoC); Complete Response (CR); Died of Disease (DoD); Disks Large homolog 2 (DLG2); Dynactin Subunit 1 (DCTN1); Echinoderm Microtubule Associated Protein-Like 4 (EML4); Entrectinib (Entre); ETS Variant Transcription Factor 6 (ETV6); Eukaryotic translation initiation factor 4B (EIF4B); Female (F); Fluorescence in situ hybridization (FISH); Follow-Up (FU); Immune Checkpoint Inhibitor (ICI); Immunohistochemistry (IHC); Infantile Fibrosarcoma (IFS); KN Motif and Ankyrin Repeat Domains 1 (KANK1); Larotrectinib (Laro); Lung Cancer (LC); Male (M); Malignant peripheral nerve sheath tumors; NACC Family Member 2 (NACC); Next-Generation Sequencing (NGS); Not Applicable (NA); Nuclear Receptor Corepressor 2 (NCOR2); Neoadjuvant (Neoadj); Ovarian Cancer (OC); Pancreas Cancer (PC); Partial Response (PR); Progressive Disease (PD); Radioactive Iodine (RAI); Radiotherapy (RT); Rho Guanine Nucleotide Exchange Factor 7 (ARHGEF7); Rho/Rac guanine nucleotide exchange factor 2 (ARHGEF2); Salivary Gland Tumor (SG); SEL1L adaptor subunit of ERAD E3 ubiquitin ligase (SEL1L); Sequestosome 1 (SQSTM1); Small Nuclear Ribonucleoprotein U1 Subunit 70 (SNRNP70); Soft Tissue Sarcoma (STS); Sperm Antigen with Calponin Homology and Coiled-Coil Domains 1 Like (SPECC1L); Stable Disease (SD); Strictosidine synthase (STR1); Surgery (Surg); Translocated Promoter Region (TPR); Targeted Therapy (TargT); Thyroid Tumor (TT); Tropomyosin 3 (TPM3); Tropomyosin 4 (TPM4); Whole-Exome Sequencing (WES); Zinc Finger CCHC-Type Containing 7 (ZCCHC7).

**Table 2:** Results of quantitative analysis of data extracted by included publications.

Included Publications		Sites of Metastases—*n* (%)		Diagnosis—*n* (%) ^†^	
Number of CR	32	LR recurrence	13 (38.2%)	NGS	36 (83.7%)
Number of CS	6	Lymph nodes	13 (38.2%)	IHC	12 (27.9%)
Year of publication (Range)	2018–2022	Lung	13 (38.2%)	FISH	8 (18.6%)
**Demographics—*n* (%)**		Bone	9 (26.5%)	RT-PCR	0 (0%)
		Liver	7 (20.6%)	**NTRKi [Drug]—*n* (%)**	
Number of patients	43	Pleura	5 (14.7%)		
Median Age	37 (<1–81)	Soft tissue	5 (14.7%)	Larotrectinib	35 (81.4%)
Adult–Children	25 (58.1%)–18 (41.9%)	Brain	3 (8.8%)	Entrectinib	7 (16.3%)
Male–Female *	19 (44.2%)–21 (48.8%)	Adrenal	3 (8.8%)	Both	1 (2.3%)
**Tumor types—*n* (%)**		Peritoneum	3 (8.8%)	**NTRKi [Line of therapy]—*n* (%) ^§^**	
		Mediastinum	2 (5.9%)		
Soft tissue sarcoma	13 (30.2%)	Kidney	2 (5.9%)	First-line	15 (34.9%)
CNS tumors	12 (27.9%)	Leptomeninges	2 (5.9%)	Second-line	15 (34.9%)
Thyroid tumors	6 (14%)	Pancreas	2 (5.9%)	Third-line	7 (16.3%)
Salivary gland tumors	4 (9.3%)	Ovarian	1 (2.9%)	Subsequent lines	3 (6.9%)
Lung tumor	2 (4.8%)	NTRK gene fusion partner—*n* (%) **		**NTRKi [Best radiological response]—*n* (%)**	
Breast cancer	1 (2.3%)				
Colon cancer	1 (2.3%)	ETV6	16 (37.2%)	Partial response	32 (74.5%)
Ovarian cancer	1 (2.3%)	TMP3	3 (7%)	Complete response	9 (20.9%)
Pancreatic tumor	1 (2.3%)	EML4	3 (7%)	Stable disease	1 (2.3%)
Thymus	1 (2.3%)	**Site of NTRK gene fusion detection—*n* (%) ^#^**		Progressive disease	1 (2.3%)
Cervix cancer	1 (2.3%)			**NTRKi [Outcomes]—*n* (%) ^##^**	
**Stage—*n* (%)**		Primary tumor	30 (69.7%)		
		Metastasis	7 (16.3%)	Alive with disease	31 (72.1%)
Metastatic	34 (79.1%)			Dead of disease	5 (11.6%)

* Not reported in 3 cases (7%). ** Most frequent NTRK gene fusion partner. ^#^ Not reported in 6 cases (14%). ^†^ Not reported in 5 cases (16.6%). ^§^ In 3 cases (7%), NTRKi were used as neoadjuvant/adjuvant treatment. ^##^ Not reported in 7 cases (16.3%). Abbreviations: Case Report (CR), Case Series (CS), Echinoderm Microtubule Associated Protein-Like 4 (EML4), ETS Variant Transcription Factor 6 (ETV6), Fluorescent in situ hybridization (FISH), Immunohistochemistry (IHC), Loco Regional (LR), DNA- or RNA-based NGS assays (NGS), NTRK inhibitors (NTRKi), Reverse Transcription–Polymerase Chain Reaction (RT-PCR), Tropomyosin 3 (TPM3).

**Table 3:** Frequencies of tumor types in the clinical trials that led to the approval of larotrectinib and entrectinib and in our virtual cohort of patients.

Tumor Types	Larotrectinib (LOXO-TRK-14001; SCOUT; NAVIGATE) [68]	Entrectinib (STARTRK-1; STARTRK-2; ALKA-372-001) [15]	Larotrectinib + Entrectinib (Virtual Cohort of CR and CS) [19,20,21,22,23,24,25,26,27,28,29,30,31,32,33,34,35,36,37,38,39,40,41,42,43,44,45,46,47,48,49,50,51,52,53,54,55,56]
Appendix cancer	1 (<1%)	-	-
Bone sarcoma	2 (1%)	-	-
Breast cancer	5 (3%)	6 (11%)	1 (2.3%)
Congenital mesoblastic nephroma	1 (<1%)	-	-
Cholangiocarcinoma	2 (1%)	1 (2%)	-
Colorectal cancer	8 (5%) *	4 (7%)	1 (2.3%) *
Cervical cancer	-	-	1 (2.3%)
Endometrial cancer	-	1 (2%)	-
Central nervous system tumor	-	-	12 (27.9%)
Hepatocellular tumor	1 (<1%)	-	-
Lung cancer	12 (8%)	10 (19%) **	2 (4.8%)
Melanoma	7 (4%)	-	-
Neuroendocrine tumor	-	3 (6%)	-
Ovarian cancer	-	1 (2%)	1 (2.3%)
Pancreas cancer	2 (1%)	3 (6%)	1 (2.3%)
Prostate cancer	1 (<1%)	-	-
Salivary gland tumor	21 (13%)	7 (13%) ^†^	4 (9.3%)
Soft tissue sarcoma	69 (44%) ^#^	13 (24%) ^§^	13 (30.2%)
Thymoma	-	-	1 (2.3%)
Thyroid cancer	26 (16%)	5 (9%)	6 (14%)
Unknown primary	1 (<1%)	-	-

* Only colon cancer. ** Only non-small cell lung cancer histology. ^†^ Only mammary analogue secretory carcinoma histology. ^§^ Including cervical adenosarcoma, dedifferentiated chondrosarcoma, endometrial stromal sarcoma, follicular dendritic cell sarcoma, gastrointestinal stromal tumor, malignant peripheral nerve sheath tumor, and sarcoma not otherwise specified. ^#^ Including infantile fibrosarcoma, gastrointestinal stromal tumor, and other.

## Data Availability

Not applicable.

## Supplementary Materials

The following supporting information can be downloaded at: https://www.mdpi.com/article/10.3390/jpm12111819/s1, PRISMA 2020 checklist (Supplementary Material S1); Risk of bias assessment for included publications (Supplementary Material S2). Ref. [80] has been cited in Supplementary Materials.

## References

[1] Tarantino P., Mazzarella L., Marra A., Trapani D., Curigliano G. (2021). The Evolving Paradigm of Biomarker Actionability: Histology-Agnosticism as a Spectrum, Rather than a Binary Quality. Cancer Treat. Rev..

[2] Tsimberidou A.M., Fountzilas E., Nikanjam M., Kurzrock R. (2020). Review of Precision Cancer Medicine: Evolution of the Treatment Paradigm. Cancer Treat. Rev..

[3] Hierro C., Matos I., Martin-Liberal J., Ochoa de Olza M., Garralda E. (2019). Agnostic-Histology Approval of New Drugs in Oncology: Are We Already There?. Clin. Cancer Res..

[4] Pestana R.C., Sen S., Hobbs B.P., Hong D.S. (2020). Histology-Agnostic Drug Development-Considering Issues beyond the Tissue. Nat. Rev. Clin. Oncol..

[5] FDA Grants Accelerated Approval to Pembrolizumab for First Tissue/Site Agnostic Indication. https://www.fda.gov/drugs/resources-information-approved-drugs/fda-grants-accelerated-approval-pembrolizumab-first-tissuesite-agnostic-indication.

[6] FDA Approves Pembrolizumab for Adults and Children with TMB-H Solid Tumors. https://www.fda.gov/drugs/drug-approvals-and-databases/fda-approves-pembrolizumab-adults-and-children-tmb-h-solid-tumors.

[7] FDA Grants Accelerated Approval to Dostarlimab-Gxly for dMMR Advanced Solid Tumors. https://www.fda.gov/drugs/resources-information-approved-drugs/fda-grants-accelerated-approval-dostarlimab-gxly-dmmr-advanced-solid-tumors.

[8] FDA Grants Accelerated Approval to Dabrafenib in Combination with Trametinib for Unresectable or Metastatic Solid Tumors with BRAF V600E Mutation. https://www.fda.gov/drugs/resources-information-approved-drugs/fda-grantsaccelerated-approval-dabrafenib-combination-trametinib-unresectable-or-metastatic-solid.

[9] FDA Approves Larotrectinib for Solid Tumors with NTRK Gene Fusions. https://www.fda.gov/drugs/fda-approves-larotrectinib-solid-tumors-ntrk-gene-fusions.

[10] FDA Approves Entrectinib for NTRK Solid Tumors and ROS-1 NSCLC. https://www.fda.gov/drugs/resourcesinformation-approved-drugs/fda-approves-entrectinib-ntrk-solid-tumors-and-ros-1-nsclc.

[11] Gatalica Z., Xiu J., Swensen J., Vranic S. (2019). Molecular Characterization of Cancers with NTRK Gene Fusions. Mod. Pathol..

[12] Solomon J.P., Benayed R., Hechtman J.F., Ladanyi M. (2019). Identifying Patients with NTRK Fusion Cancer. Ann. Oncol..

[13] Cocco E., Scaltriti M., Drilon A. (2018). NTRK Fusion-Positive Cancers and TRK Inhibitor Therapy. Nat. Rev. Clin. Oncol..

[14] Drilon A., Laetsch T.W., Kummar S., DuBois S.G., Lassen U.N., Demetri G.D., Nathenson M., Doebele R.C., Farago A.F., Pappo A.S. (2018). Efficacy of Larotrectinib in TRK Fusion-Positive Cancers in Adults and Children. N. Engl. J. Med..

[15] Doebele R.C., Drilon A., Paz-Ares L., Siena S., Shaw A.T., Farago A.F., Blakely C.M., Seto T., Cho B.C., Tosi D. (2020). Entrectinib in Patients with Advanced or Metastatic NTRK Fusion-Positive Solid Tumours: Integrated Analysis of Three Phase 1-2 Trials. Lancet Oncol..

[16] Malone E.R., Oliva M., Sabatini P.J.B., Stockley T.L., Siu L.L. (2020). Molecular Profiling for Precision Cancer Therapies. Genome Med..

[17] PRISMA—Transparent Reporting of Systematic Reviews and Meta-Analyses. http://prisma-statement.org/.

[18] Murad M.H., Sultan S., Haffar S., Bazerbachi F. (2018). Methodological Quality and Synthesis of Case Series and Case Reports. BMJ Evid. Based Med..

[19] Landman Y., Ilouze M., Wein S., Neiman V., Yerushalmi R., Yakimov M., Ku N., Schrock A.B., Ali S., Peled N. (2018). Rapid Response to Larotrectinib (LOXO-101) in an Adult Chemotherapy-Naive Patients with Advanced Triple-Negative Secretory Breast Cancer Expressing ETV6-NTRK3 Fusion. Clin. Breast Cancer.

[20] Ziegler D.S., Wong M., Mayoh C., Kumar A., Tsoli M., Mould E., Tyrrell V., Khuong-Quang D.-A., Pinese M., Gayevskiy V. (2018). Brief Report: Potent Clinical and Radiological Response to Larotrectinib in TRK Fusion-Driven High-Grade Glioma. Br. J. Cancer.

[21] Wong D.D., Vargas A.C., Bonar F., Maclean F., Kattampallil J., Stewart C., Sulaiman B., Santos L., Gill A.J. (2020). NTRK-Rearranged Mesenchymal Tumours: Diagnostic Challenges, Morphological Patterns and Proposed Testing Algorithm. Pathology.

[22] Hochmair M.J., Setinek U., Krenbek D., Fazekas A., Illini O., Weinlinger C., Draxler H., Marcher M., Valipour A., Müllauer L. (2020). Rapid Clinical and Radiologic Responses with Larotrectinib Treatment in a Patient with TRK-Fusion-Positive Metastatic Lung Cancer. Clin. Lung Cancer.

[23] Alharbi M., Mobark N.A., Balbaid A.A.O., Alanazi F.A., Aljabarat W.A.R., Bakhsh E.A., Ramkissoon S.H., Abedalthagafi M. (2020). Regression of ETV6-NTRK3 Infantile Glioblastoma After First-Line Treatment with Larotrectinib. JCO Precis. Oncol..

[24] Mayr L., Guntner A.S., Madlener S., Schmook M.T., Peyrl A., Azizi A.A., Dieckmann K., Reisinger D., Stepien N.M., Schramm K. (2020). Cerebrospinal Fluid Penetration and Combination Therapy of Entrectinib for Disseminated ROS1/NTRK-Fusion Positive Pediatric High-Grade Glioma. J. Pers. Med..

[25] Walter A.W., Kandula V.V.R., Shah N. (2020). Larotrectinib Imaging Response in Low-Grade Glioma. Pediatr. Blood Cancer.

[26] Salame H., Mckey R., Ballout M., Saad W. (2021). The First Reported Case of Neurotrophic Tyrosine Receptor Kinase Fusion-Positive Thymoma Treated Successfully with Entrectinib. Cureus.

[27] Zhang L., Liu H., Tian Y., Wang H., Yang X. (2021). A Novel NCOR2-NTRK1 Fusion Detected in a Patient of Lung Adenocarcinoma and Response to Larotrectinib: A Case Report. BMC Pulm. Med..

[28] Gupta M., Sherrow C., Krone M.E., Blais E.M., Pishvaian M.J., Petricoin E.F., Matrisian L.M., DeArbeloa P., Gregory G., Brown A. (2021). Targeting the NTRK Fusion Gene in Pancreatic Acinar Cell Carcinoma: A Case Report and Review of the Literature. J. Natl. Compr. Canc. Netw..

[29] Percy C., Schubert T., Galant C., Kirchgesner T., Mazzeo F. (2021). Larotrectinib in a NTRK-Rearranged Soft Tissue Sarcoma in the Neoadjuvant Setting: A Case Report. Clin. Case Rep..

[30] Munkhdelger J., Shimooka T., Koyama Y., Ikeda S., Mikami Y., Fukuoka J., Hori T., Bychkov A. (2021). Basaloid Squamous Cell Carcinoma of the Uterine Cervix: Report of a Case with Molecular Analysis. Int. J. Surg. Pathol..

[31] Pircher M., Briner H.R., Bonomo M., Horcic M., Petrausch U., Helbling D., Winder T. (2021). Mixed Response and Mechanisms of Resistance to Larotrectinib in Metastatic Carcinoma Ex Pleomorphic Adenoma of the Parotid Harboring an NTRK2 Fusion: A Case Report. Medicine (Baltimore).

[32] Pitoia F. (2021). Complete Response to Larotrectinib Treatment in a Patient with Papillary Thyroid Cancer Harboring an ETV6-NTRK3 Gene Fusion. Clin. Case Rep..

[33] Shepherd D.J., Miller T.E., Forst D.A., Jones P., Nardi V., Martinez-Lage M., Stemmer-Rachamimov A., Gonzalez R.G., Iafrate A.J., Ritterhouse L.L. (2021). Mosaicism for Rec.ceptor Tyrosine Kinase Activation in a Glioblastoma Involving Both PDGFRA Amplification and NTRK2 Fusion. Oncologist.

[34] Wagner F., Greim R., Krebs K., Luebben F., Dimmler A. (2021). Characterization of an ETV6-NTRK3 Rearrangement with Unusual, but Highly Significant FISH Signal Pattern in a Secretory Carcinoma of the Salivary Gland: A Case Report. Diagn. Pathol..

[35] Boyer J., Birzu C., Bielle F., Goulas C., Savatovsky J., Karachi C., Idbaih A. (2021). Dramatic Response of STRN-NTRK-Fused Malignant Glioneuronal Tumor to Larotrectinib in Adult. Neuro Oncol..

[36] Corral Sánchez M.D., Galán Gómez V., Sastre Urgelles A., Plaza López de Sabando D., Rubio Aparicio P., Martínez Martínez L., Alonso Gamarra E., Pozo Kreilinger J.J., Regojo Zapata R.M., López Gutiérrez J.C. (2021). Treatment of Infantile Fibrosarcoma Associated to an Abdominal Aortic Aneurysm with Larotrectinib: A Case Report. Pediatr. Hematol. Oncol..

[37] Goh X.N., Seng M.S.-F., Loh A.H.P., Gupta A., Chang K.T.E., Iyer P. (2021). Larotrectinib Followed by Selitrectinib in a Novel DCTN1-NTRK1 Fusion Undifferentiated Pleomorphic Sarcoma. J. Oncol. Pharm. Pract..

[38] Carter-Febres M., Schneller N., Fair D., Solomon D., Perry A., Roy A., Linscott L., Alashari M., Kestle J.R., Bruggers C.S. (2021). Adjuvant Maintenance Larotrectinib Therapy in 2 Children with NTRK Fusion-Positive High-Grade Cancers. J. Pediatr. Hematol. Oncol.

[39] Slomovic A., Amaral T., Lobko I., Siegel D.N., Goldfisher R., Kessel R., Levy C.F. (2021). Comment on: A Newborn with a Large NTRK Fusion Positive Infantile Fibrosarcoma Successfully Treated with Larotrectinib. Pediatr. Blood Cancer.

[40] Waters T.W., Moore S.A., Sato Y., Dlouhy B.J., Sato M. (2021). Refractory Infantile High-Grade Glioma Containing TRK-Fusion Responds to Larotrectinib. Pediatr. Blood Cancer.

[41] Mangum R., Reuther J., Bertrand K.C., Chandramohan R., Kukreja M.K., Paulino A.C., Muzny D., Hu J., Gibbs R.A., Curry D.J. (2021). Durable Response to Larotrectinib in a Child with Histologic Diagnosis of Recurrent Disseminated Ependymoma Discovered to Harbor an NTRK2 Fusion: The Impact of Integrated Genomic Profiling. JCO Precis. Oncol..

[42] Endo Y., Watanabe T., Saito M., Saito K., Suzuki R., Sano H., Natori Y., Sasaki E., Ueda M., Kamo N. (2022). A Rare Case of Recurrent Ovarian Cancer with TPM3-NTRK1 Gene Rearrangement: A Case Report. Mol. Clin. Oncol..

[43] Ernst M.S., Lysack J.T., Hyrcza M.D., Chandarana S.P., Hao D. (2022). TRK Inhibition with Entrectinib in Metastatic Salivary Secretory Carcinoma (SC): A Case Report. Curr. Oncol..

[44] Recine F., De Vita A., Fausti V., Pieri F., Bongiovanni A., Franchini E., Casadei R., Falasconi M.C., Oboldi D., Matteucci F. (2021). Case Report: Adult NTRK-Rearranged Spindle Cell Neoplasm: Early Tumor Shrinkage in a Case with Bone and Visceral Metastases Treated with Targeted Therapy. Front. Oncol..

[45] Bill R., Deschler D.G., Pittet M.J., Pai S.I., Sadow P.M., Park J.C. (2022). Diagnostic Challenges and Successful Organ-Preserving Therapy in a Case of Secretory Carcinoma of Minor Salivary Glands. Cancer Rep. (Hoboken).

[46] Bargas S., Mc Leer A., Mondet J., Chabre O., Laramas M. (2022). An Impressive Response with Larotrectinib in a Patient with a Papillary Thyroid Carcinoma Harboring an SQSTM1-NTRK1 Fusion. Eur. J. Endocrinol..

[47] Kasi P.M., Afghan M.K., Bellizzi A.M., Chan C.H. (2022). Larotrectinib in Mismatch-Repair-Deficient TRK Fusion-Positive Metastatic Colon Cancer after Progression on Immunotherapy. Cureus.

[48] Saliba M., Mohanty A.S., Ho A.L., Drilon A., Dogan S. (2022). Secretory Carcinoma of the Thyroid in a 49-Year-Old Man Treated with Larotrectinib: Protracted Clinical Course of Disease Despite the High-Grade Histologic Features. Head Neck Pathol..

[49] Lapeña L.M., Caldas M.C.S., Ramírez C., Basilio M.S., Junco P.T., Rodríguez-Laguna L., Martínez-González V., Marín-Manzano E., Perez-Martinez A., Lopez-Gutierrez J.C. (2022). Larotrectinib as an Effective Therapy in Congenital Infantile Fibrosarcoma: Report of Two Cases. Eur. J. Pediatr. Surg. Rep..

[50] Groussin L., Theodon H., Bessiene L., Bricaire L., Bonnet-Serrano F., Cochand-Priollet B., Leroy K., Garinet S., Pasmant E., Zerbit J. (2022). Redifferentiating Effect of Larotrectinib in NTRK-Rearranged Advanced Radioactive-Iodine Refractory Thyroid Cancer. Thyroid.

[51] Grogan P.T., Deming D.A., Helgager J., Ruszkiewicz T., Baskaya M.K., Howard S.P., Robins H.I. (2022). Entrectinib Demonstrates Prolonged Efficacy in an Adult Case of Radiation-Refractory NTRK Fusion Glioblastoma. Neurooncol. Adv..

[52] Kobayashi H., Makise N., Shinozaki-Ushiku A., Zhang L., Ishibashi Y., Ikegami M., Tsuda Y., Kohsaka S., Ushiku T., Oda K. (2022). Dramatic Response to Entrectinib in a Patient with Malignant Peripheral Nerve Sheath Tumor Harboring Novel SNRNP70-NTRK3 Fusion Gene. Genes Chromosom. Cancer.

[53] König D., Hench J., Frank S., Dima L., Bratic Hench I., Läubli H. (2022). Larotrectinib Response in NTRK3 Fusion-Driven Diffuse High-Grade Glioma. Pharmacology.

[54] Olsen M.R., Denu R.A., Lyon J.B., Gulliver J.M., Capitini C.M., DeSantes K.B. (2022). Undifferentiated and Unresectable Sarcoma with NTRK3-Fusion in a Pediatric Patient Treated with Larotrectinib and Proton Beam Radiotherapy. J. Pediatr. Hematol. Oncol..

[55] Mançano B.M., Dos Reis M.B., Moreno D.A., de Paula F.E., de Almeida Junior C.R., Cavalcante C.E.B., Zanon M.F., Santana I.V.V., Matsushita M., de M. (2022). A Unique Case Report of Infant-Type Hemispheric Glioma (Gliosarcoma Subtype) with TPR-NTRK1 Fusion Treated with Larotrectinib. Pathobiology.

[56] Di Ruscio V., Carai A., Del Baldo G., Vinci M., Cacchione A., Miele E., Rossi S., Antonelli M., Barresi S., Caulo M. (2022). Molecular Landscape in Infant High-Grade Gliomas: A Single Center Experience. Diagnostics.

[57] Kurozumi K., Fujii K., Washio K., Ishida J., Otani Y., Sudo T., Tahara M., Ichimura K., Ennishi D., Date I. (2022). Response to Entrectinib in a Malignant Glioneuronal Tumor with ARHGEF2-NTRK Fusion. Neurooncol. Adv..

[58] Iannantuono G.M., Torino F., Rosenfeld R., Guerriero S., Carlucci M., Sganga S., Capotondi B., Riondino S., Roselli M. (2022). The Role of Histology-Agnostic Drugs in the Treatment of Metastatic Castration-Resistant Prostate Cancer. Int. J. Mol. Sci..

[59] Looney A.-M., Nawaz K., Webster R.M. (2020). Tumour-Agnostic Therapies. Nat. Rev. Drug Discov..

[60] Amatu A., Sartore-Bianchi A., Siena S. (2016). NTRK Gene Fusions as Novel Targets of Cancer Therapy across Multiple Tumour Types. ESMO Open.

[61] Liu F., Wei Y., Zhang H., Jiang J., Zhang P., Chu Q. (2022). NTRK Fusion in Non-Small Cell Lung Cancer: Diagnosis, Therapy, and TRK Inhibitor Resistance. Front. Oncol..

[62] Vaishnavi A., Capelletti M., Le A.T., Kako S., Butaney M., Ercan D., Mahale S., Davies K.D., Aisner D.L., Pilling A.B. (2013). Oncogenic and Drug-Sensitive NTRK1 Rearrangements in Lung Cancer. Nat. Med..

[63] Rosen E.Y., Goldman D.A., Hechtman J.F., Benayed R., Schram A.M., Cocco E., Shifman S., Gong Y., Kundra R., Solomon J.P. (2020). TRK Fusions Are Enriched in Cancers with Uncommon Histologies and the Absence of Canonical Driver Mutations. Clin. Cancer Res..

[64] Forsythe A., Zhang W., Phillip Strauss U., Fellous M., Korei M., Keating K. (2020). A Systematic Review and Meta-Analysis of Neurotrophic Tyrosine Receptor Kinase Gene Fusion Frequencies in Solid Tumors. Ther. Adv. Med. Oncol..

[65] Westphalen C.B., Krebs M.G., Le Tourneau C., Sokol E.S., Maund S.L., Wilson T.R., Jin D.X., Newberg J.Y., Fabrizio D., Veronese L. (2021). Genomic Context of NTRK1/2/3 Fusion-Positive Tumours from a Large Real-World Population. NPJ Precis. Oncol..

[66] Hechtman J.F. (2022). NTRK Insights: Best Practices for Pathologists. Mod. Pathol..

[67] Marchiò C., Scaltriti M., Ladanyi M., Iafrate A.J., Bibeau F., Dietel M., Hechtman J.F., Troiani T., López-Rios F., Douillard J.-Y. (2019). ESMO Recommendations on the Standard Methods to Detect NTRK Fusions in Daily Practice and Clinical Research. Ann. Oncol..

[68] Hong D.S., DuBois S.G., Kummar S., Farago A.F., Albert C.M., Rohrberg K.S., van Tilburg C.M., Nagasubramanian R., Berlin J.D., Federman N. (2020). Larotrectinib in Patients with TRK Fusion-Positive Solid Tumours: A Pooled Analysis of Three Phase 1/2 Clinical Trials. Lancet Oncol..

[69] Drilon A.E., Hong D.S., van Tilburg C.M., Doz F., Tan D.S.W., Kummar S., Lin J.J., McDermott R.S., Zwaan C.M., Norenberg R. (2022). Long-Term Efficacy and Safety of Larotrectinib in a Pooled Analysis of Patients with Tropomyosin Receptor Kinase (TRK) Fusion Cancer. JCO.

[70] Iannantuono G.M., Riondino S., Sganga S., Roselli M., Torino F. (2022). Activity of ALK Inhibitors in Renal Cancer with ALK Alterations: A Systematic Review. Int. J. Mol. Sci..

[71] Gambella A., Senetta R., Collemi G., Vallero S.G., Monticelli M., Cofano F., Zeppa P., Garbossa D., Pellerino A., Rudà R. (2020). NTRK Fusions in Central Nervous System Tumors: A Rare, but Worthy Target. Int. J. Mol. Sci..

[72] NCCN Clinical Practice Guidelines in Oncology (NCCN Guidelines^®^) Central Nervous System Cancers. https://www.nccn.org/professionals/physician_gls/pdf/cns.pdf.

[73] Doz F., van Tilburg C.M., Geoerger B., Højgaard M., Øra I., Boni V., Capra M., Chisholm J., Chung H.C., DuBois S.G. (2022). Efficacy and Safety of Larotrectinib in TRK Fusion-Positive Primary Central Nervous System Tumors. Neuro Oncol..

[74] Li M.M., Datto M., Duncavage E.J., Kulkarni S., Lindeman N.I., Roy S., Tsimberidou A.M., Vnencak-Jones C.L., Wolff D.J., Younes A. (2017). Standards and Guidelines for the Interpretation and Reporting of Sequence Variants in Cancer: A Joint Consensus Recommendation of the Association for Molecular Pathology, American Society of Clinical Oncology, and College of American Pathologists. J. Mol. Diagn..

[75] Awada A., Berghmans T., Clement P.M., Cuppens K., De Wilde B., Machiels J.-P., Pauwels P., Peeters M., Rottey S., Van Cutsem E. (2022). Belgian Expert Consensus for Tumor-Agnostic Treatment of NTRK Gene Fusion-Driven Solid Tumors with Larotrectinib. Crit. Rev. Oncol. Hematol..

[76] Registry for Molecular Testing, Treatment and Outcome of Patients with Solid Tumors Harboring a NTRK Gene Fusion (REALTRK). https://clinicaltrials.gov/ct2/show/NCT04557813.

[77] Real Word European Registry of NTRK Fusions and Other Rare Actionable Fusions (TRacKING) (TRacKING). https://www.clinicaltrials.gov/ct2/show/NCT04921553.

[78] Smith C.M., Gilbert E.B., Riordan P.A., Helmke N., von Isenburg M., Kincaid B.R., Shirey K.G. (2021). COVID-19-Associated Psychosis: A Systematic Review of Case Reports. Gen. Hosp. Psychiatry.

[79] Iannantuono G.M., Strigari L., Roselli M., Torino F. (2021). A Scoping Review on the “Burned out” or “Burnt out” Testicular Cancer: When a Rare Phenomenon Deserves More Attention. Crit. Rev. Oncol. Hematol..

[80] Page M.J., McKenzie J.E., Bossuyt P.M., Boutron I., Hoffmann T.C., Mulrow C.D., Shamseer L., Tetzlaff J.M., Akl E.A., Brennan E.A. (2021). The PRISMA 2020 statement: An updated guideline for reporting systematic reviews. BMJ.

