# Supplementary material for: NTRK Gene Fusions in Solid Tumors and TRK Inhibitors: A Systematic Review of Case Reports and Case Series
Source: J Pers Med. 2022 Nov 2;12(11):1819. doi: 10.3390/jpm12111819 (PMC9695027; doi:10.3390/jpm12111819)
Supplement: Supplementary file 1 [file jpm-12-01819-s001.zip › Supplementary Material 2_20221025.pdf]

Tool for evaluating the methodological quality of case reports and case series

Murad et al. BMJ Evid Based Med. 2018

Landman Y, Ilouze M, Wein S et al. Rapid Response to Larotrectinib (LOXO-101) in an Adult Chemotherapy-Naive Patients With Advanced Triple-Negative Secretory Breast Cancer Expressing ETV6-NTRK3 Fusion. Clin Breast Cancer. 2018 Jun;18(3):e267-e270. doi: 10.1016/j.clbc.2017.11.017.

**Domains**	**Leading explanatory questions**	**Answer**
Selection	**1.** Does the patient(s) represent(s) the whole experience of the investigator (centre) or is the selection method unclear to the extent that other patients with similar presentation may not have been reported?	1
Ascertainment	**2.** Was the exposure adequately ascertained?	1
	**3.** Was the outcome adequately ascertained?	1
Causality	**4.** Were other alternative causes that may explain the observation ruled out?	0
	**5.** Was there a challenge/rechallenge phenomenon?	0
	**6.** Was there a dose–response effect?	1
	**7.** Was follow-up long enough for outcomes to occur?	1
Reporting	**8.** Is the case(s) described with sufficient details to allow other investigators to replicate the research or to allow practitioners make inferences related to their own practice?	1

Tool for evaluating the methodological quality of case reports and case series

Murad et al. BMJ Evid Based Med. 2018

Ziegler DS, Wong M, Mayoh C et al. Brief Report: Potent clinical and radiological response to larotrectinib in TRK fusion-driven high-grade glioma. doi: 10.1038/s41416-018-0251-2.

Domains	Leading explanatory questions	Answer
Selection	**1.** Does the patient(s) represent(s) the whole experience of the investigator (centre) or is the selection method unclear to the extent that other patients with similar presentation may not have been reported?	0
Ascertainment	**2.** Was the exposure adequately ascertained?	1
	**3.** Was the outcome adequately ascertained?	1
Causality	**4.** Were other alternative causes that may explain the observation ruled out?	1
	**5.** Was there a challenge/rechallenge phenomenon?	0
	**6.** Was there a dose–response effect?	0
	**7.** Was follow-up long enough for outcomes to occur?	1
Reporting	**8.** Is the case(s) described with sufficient details to allow other investigators to replicate the research or to allow practitioners make inferences related to their own practice?	1

Tool for evaluating the methodological quality of case reports and case series

Murad et al. BMJ Evid Based Med. 2018

Wong DD, Vargas AC, Bonar F et al. NTRK-rearranged mesenchymal tumours: diagnostic challenges, morphological patterns and proposed testing algorithm. doi: 10.1016/j.pathol.2020.02.004.

Domains	Leading explanatory questions	Answer
Selection	**1.** Does the patient(s) represent(s) the whole experience of the investigator (centre) or is the selection method unclear to the extent that other patients with similar presentation may not have been reported?	1
Ascertainment	**2.** Was the exposure adequately ascertained?	0
	**3.** Was the outcome adequately ascertained?	0
Causality	**4.** Were other alternative causes that may explain the observation ruled out?	0
	**5.** Was there a challenge/rechallenge phenomenon?	0
	**6.** Was there a dose–response effect?	0
	**7.** Was follow-up long enough for outcomes to occur?	0
Reporting	**8.** Is the case(s) described with sufficient details to allow other investigators to replicate the research or to allow practitioners make inferences related to their own practice?	0

Tool for evaluating the methodological quality of case reports and case series

Murad et al. BMJ Evid Based Med. 2018

Hochmair MJ, Setinek U, Krenbek D et al. Rapid Clinical and Radiologic Responses With Larotrectinib Treatment in a Patient With TRK-Fusion-Positive Metastatic Lung Cancer. doi: 10.1016/j.cllc.2019.11.010.

Domains	Leading explanatory questions	Answer
Selection	**1.** Does the patient(s) represent(s) the whole experience of the investigator (centre) or is the selection method unclear to the extent that other patients with similar presentation may not have been reported?	1
Ascertainment	**2.** Was the exposure adequately ascertained?	1
	**3.** Was the outcome adequately ascertained?	0
Causality	**4.** Were other alternative causes that may explain the observation ruled out?	1
	**5.** Was there a challenge/rechallenge phenomenon?	0
	**6.** Was there a dose–response effect?	0
	**7.** Was follow-up long enough for outcomes to occur?	1
Reporting	**8.** Is the case(s) described with sufficient details to allow other investigators to replicate the research or to allow practitioners make inferences related to their own practice?	1

Tool for evaluating the methodological quality of case reports and case series

Murad et al. BMJ Evid Based Med. 2018

Alharbi M, Mobark NA, Balbaid AAO, et al. Regression of ETV6-NTRK3 Infantile Glioblastoma After First-Line Treatment With Larotrectinib. JCO Precis Oncol. 2020 Jun 30;4:PO.20.00017. doi: 10.1200/PO.20.00017.

Domains	Leading explanatory questions	Answer
Selection	**1.** Does the patient(s) represent(s) the whole experience of the investigator (centre) or is the selection method unclear to the extent that other patients with similar presentation may not have been reported?	1
Ascertainment	**2.** Was the exposure adequately ascertained?	1
	**3.** Was the outcome adequately ascertained?	1
Causality	**4.** Were other alternative causes that may explain the observation ruled out?	0
	**5.** Was there a challenge/rechallenge phenomenon?	0
	**6.** Was there a dose–response effect?	0
	**7.** Was follow-up long enough for outcomes to occur?	1
Reporting	**8.** Is the case(s) described with sufficient details to allow other investigators to replicate the research or to allow practitioners make inferences related to their own practice?	1

Tool for evaluating the methodological quality of case reports and case series

Murad et al. BMJ Evid Based Med. 2018

Mayr L, Guntner AS, Madlener S et al. Cerebrospinal Fluid Penetration and Combination Therapy of Entrectinib for Disseminated ROS1/NTRK-Fusion Positive Pediatric High-Grade Glioma. J Pers Med. 2020 Dec 18;10(4):290. doi: 10.3390/jpm10040290.

Domains	Leading explanatory questions	Answer
Selection	**1.** Does the patient(s) represent(s) the whole experience of the investigator (centre) or is the selection method unclear to the extent that other patients with similar presentation may not have been reported?	1
Ascertainment	**2.** Was the exposure adequately ascertained?	1
	**3.** Was the outcome adequately ascertained?	0
Causality	**4.** Were other alternative causes that may explain the observation ruled out?	1
	**5.** Was there a challenge/rechallenge phenomenon?	0
	**6.** Was there a dose–response effect?	0
	**7.** Was follow-up long enough for outcomes to occur?	1
Reporting	**8.** Is the case(s) described with sufficient details to allow other investigators to replicate the research or to allow practitioners make inferences related to their own practice?	0

Tool for evaluating the methodological quality of case reports and case series

Murad et al. BMJ Evid Based Med. 2018

Walter AW, Kandula VVR, Shah N. Larotrectinib imaging response in low-grade glioma. Pediatr Blood Cancer. 2020 Jan;67(1):e28002. doi: 10.1002/pbc.28002.

Domains	Leading explanatory questions	Answer
Selection	**1.** Does the patient(s) represent(s) the whole experience of the investigator (centre) or is the selection method unclear to the extent that other patients with similar presentation may not have been reported?	1
Ascertainment	**2.** Was the exposure adequately ascertained?	0
	**3.** Was the outcome adequately ascertained?	1
Causality	**4.** Were other alternative causes that may explain the observation ruled out?	1
	**5.** Was there a challenge/rechallenge phenomenon?	1
	**6.** Was there a dose–response effect?	0
	**7.** Was follow-up long enough for outcomes to occur?	0
Reporting	**8.** Is the case(s) described with sufficient details to allow other investigators to replicate the research or to allow practitioners make inferences related to their own practice?	1

Tool for evaluating the methodological quality of case reports and case series

Murad et al. BMJ Evid Based Med. 2018

Salame H, Mckey R, Ballout et al. The First Reported Case of Neurotrophic Tyrosine Receptor Kinase Fusion-Positive Thymoma Treated Successfully With Entrectinib. Cureus. 2021 Dec 21;13(12):e20588. doi: 10.7759/cureus.20588.

**Domains**	**Leading explanatory questions**	**Answer**
Selection	**1.** Does the patient(s) represent(s) the whole experience of the investigator (centre) or is the selection method unclear to the extent that other patients with similar presentation may not have been reported?	1
Ascertainment	**2.** Was the exposure adequately ascertained?	1
	**3.** Was the outcome adequately ascertained?	0
Causality	**4.** Were other alternative causes that may explain the observation ruled out?	0
	**5.** Was there a challenge/rechallenge phenomenon?	0
	**6.** Was there a dose–response effect?	1
	**7.** Was follow-up long enough for outcomes to occur?	1
Reporting	**8.** Is the case(s) described with sufficient details to allow other investigators to replicate the research or to allow practitioners make inferences related to their own practice?	1

Tool for evaluating the methodological quality of case reports and case series
Murad et al. BMJ Evid Based Med. 2018

Zhang L, Liu H, Tian Y et al. A novel NCOR2-NTRK1 fusion detected in a patient of lung adenocarcinoma and response to larotrectinib: a case report. BMC Pulm Med. 2021 Apr 17;21(1):125. doi: 10.1186/s12890-021-01490-x.

Domains	Leading explanatory questions	Answer
Selection	**1.** Does the patient(s) represent(s) the whole experience of the investigator (centre) or is the selection method unclear to the extent that other patients with similar presentation may not have been reported?	1
Ascertainment	**2.** Was the exposure adequately ascertained?	1
	**3.** Was the outcome adequately ascertained?	1
Causality	**4.** Were other alternative causes that may explain the observation ruled out?	0
	**5.** Was there a challenge/rechallenge phenomenon?	0
	**6.** Was there a dose–response effect?	1
	**7.** Was follow-up long enough for outcomes to occur?	1
Reporting	**8.** Is the case(s) described with sufficient details to allow other investigators to replicate the research or to allow practitioners make inferences related to their own practice?	1

Tool for evaluating the methodological quality of case reports and case series

Murad et al. BMJ Evid Based Med. 2018

Gupta M, Sherrow C, Krone ME et al. Targeting the NTRK Fusion Gene in Pancreatic Acinar Cell Carcinoma: A Case Report and Review of the Literature. J Natl Compr Canc Netw. 2021 Jan 6;19(1):10-15. doi: 10.6004/jnccn.2020.7641.

Domains	Leading explanatory questions	Answer
Selection	**1.** Does the patient(s) represent(s) the whole experience of the investigator (centre) or is the selection method unclear to the extent that other patients with similar presentation may not have been reported?	1
Ascertainment	**2.** Was the exposure adequately ascertained?	1
	**3.** Was the outcome adequately ascertained?	1
Causality	**4.** Were other alternative causes that may explain the observation ruled out?	0
	**5.** Was there a challenge/rechallenge phenomenon?	0
	**6.** Was there a dose–response effect?	1
	**7.** Was follow-up long enough for outcomes to occur?	1
Reporting	**8.** Is the case(s) described with sufficient details to allow other investigators to replicate the research or to allow practitioners make inferences related to their own practice?	1

Tool for evaluating the methodological quality of case reports and case series

Murad et al. BMJ Evid Based Med. 2018

Percy C, Schubert T, Galant C et al. Larotrectinib in a NTRK-rearranged soft tissue sarcoma in the neoadjuvant setting: A case report. Clin Case Rep. 2021 Feb 9;9(3):1694-1698. doi: 10.1002/ccr3.3878.

Domains	Leading explanatory questions	Answer
Selection	**1.** Does the patient(s) represent(s) the whole experience of the investigator (centre) or is the selection method unclear to the extent that other patients with similar presentation may not have been reported?	1
Ascertainment	**2.** Was the exposure adequately ascertained?	1
	**3.** Was the outcome adequately ascertained?	1
Causality	**4.** Were other alternative causes that may explain the observation ruled out?	0
	**5.** Was there a challenge/rechallenge phenomenon?	0
	**6.** Was there a dose–response effect?	1
	**7.** Was follow-up long enough for outcomes to occur?	1
Reporting	**8.** Is the case(s) described with sufficient details to allow other investigators to replicate the research or to allow practitioners make inferences related to their own practice?	1

Tool for evaluating the methodological quality of case reports and case series

Murad et al. BMJ Evid Based Med. 2018

Munkhdelger J, Shimooka T, Koyama Y et al. Basaloid Squamous Cell Carcinoma of the Uterine Cervix: Report of a Case With Molecular Analysis. Int J Surg Pathol. 2021 Oct;29(7):770-774. doi: 10.1177/1066896921997132.

Domains	Leading explanatory questions	Answer
Selection	**1.** Does the patient(s) represent(s) the whole experience of the investigator (centre) or is the selection method unclear to the extent that other patients with similar presentation may not have been reported?	1
Ascertainment	**2.** Was the exposure adequately ascertained?	1
	**3.** Was the outcome adequately ascertained?	0
Causality	**4.** Were other alternative causes that may explain the observation ruled out?	0
	**5.** Was there a challenge/rechallenge phenomenon?	0
	**6.** Was there a dose–response effect?	1
	**7.** Was follow-up long enough for outcomes to occur?	1
Reporting	**8.** Is the case(s) described with sufficient details to allow other investigators to replicate the research or to allow practitioners make inferences related to their own practice?	1

Tool for evaluating the methodological quality of case reports and case series

Murad et al. BMJ Evid Based Med. 2018

Pircher M, Briner HR, Bonomo M et al. Mixed response and mechanisms of resistance to larotrectinib in metastatic carcinoma ex pleomorphic adenoma of the parotid harboring an NTRK2 fusion: A case report. Medicine (Baltimore). 2021 Jan 29;100(4):e24463. doi: 10.1097/MD.0000000000024463.

Domains	Leading explanatory questions	Answer
Selection	**1.** Does the patient(s) represent(s) the whole experience of the investigator (centre) or is the selection method unclear to the extent that other patients with similar presentation may not have been reported?	1
Ascertainment	**2.** Was the exposure adequately ascertained?	1
	**3.** Was the outcome adequately ascertained?	1
Causality	**4.** Were other alternative causes that may explain the observation ruled out?	0
	**5.** Was there a challenge/rechallenge phenomenon?	0
	**6.** Was there a dose–response effect?	1
	**7.** Was follow-up long enough for outcomes to occur?	0
Reporting	**8.** Is the case(s) described with sufficient details to allow other investigators to replicate the research or to allow practitioners make inferences related to their own practice?	1

Tool for evaluating the methodological quality of case reports and case series

Murad et al. BMJ Evid Based Med. 2018

Pitoia F. Complete response to larotrectinib treatment in a patient with papillary thyroid cancer harboring an ETV6-NTRK3 gene fusion. Clin Case Rep. 2021 Feb 20;9(4):1905-1912. doi: 10.1002/ccr3.3900.

Domains	Leading explanatory questions	Answer
Selection	**1.** Does the patient(s) represent(s) the whole experience of the investigator (centre) or is the selection method unclear to the extent that other patients with similar presentation may not have been reported?	1
Ascertainment	**2.** Was the exposure adequately ascertained?	1
	**3.** Was the outcome adequately ascertained?	0
Causality	**4.** Were other alternative causes that may explain the observation ruled out?	0
	**5.** Was there a challenge/rechallenge phenomenon?	0
	**6.** Was there a dose–response effect?	1
	**7.** Was follow-up long enough for outcomes to occur?	0
Reporting	**8.** Is the case(s) described with sufficient details to allow other investigators to replicate the research or to allow practitioners make inferences related to their own practice?	1

Tool for evaluating the methodological quality of case reports and case series

Murad et al. BMJ Evid Based Med. 2018

Shepherd DJ, Miller TE, Forst DA et al. Mosaicism for Receptor Tyrosine Kinase Activation in a Glioblastoma Involving Both PDGFRA Amplification and NTRK2 Fusion. Oncologist. 2021 Nov;26(11):919-924. doi: 10.1002/onco.13835.

Domains	Leading explanatory questions	Answer
Selection	**1.** Does the patient(s) represent(s) the whole experience of the investigator (centre) or is the selection method unclear to the extent that other patients with similar presentation may not have been reported?	1
Ascertainment	**2.** Was the exposure adequately ascertained?	1
	**3.** Was the outcome adequately ascertained?	1
Causality	**4.** Were other alternative causes that may explain the observation ruled out?	0
	**5.** Was there a challenge/rechallenge phenomenon?	0
	**6.** Was there a dose–response effect?	0
	**7.** Was follow-up long enough for outcomes to occur?	0
Reporting	**8.** Is the case(s) described with sufficient details to allow other investigators to replicate the research or to allow practitioners make inferences related to their own practice?	1

Tool for evaluating the methodological quality of case reports and case series

Murad et al. BMJ Evid Based Med. 2018

Wagner F, Greim R, Krebs K et al. Characterization of an ETV6-NTRK3 rearrangement with unusual, but highly significant FISH signal pattern in a secretory carcinoma of the salivary gland: a case report. Diagn Pathol. 2021 Aug 9;16(1):73. doi: 10.1186/s13000-021-01133-z.

Domains	Leading explanatory questions	Answer
Selection	**1.** Does the patient(s) represent(s) the whole experience of the investigator (centre) or is the selection method unclear to the extent that other patients with similar presentation may not have been reported?	1
Ascertainment	**2.** Was the exposure adequately ascertained?	1
	**3.** Was the outcome adequately ascertained?	1
Causality	**4.** Were other alternative causes that may explain the observation ruled out?	0
	**5.** Was there a challenge/rechallenge phenomenon?	0
	**6.** Was there a dose–response effect?	1
	**7.** Was follow-up long enough for outcomes to occur?	0
Reporting	**8.** Is the case(s) described with sufficient details to allow other investigators to replicate the research or to allow practitioners make inferences related to their own practice?	1

Tool for evaluating the methodological quality of case reports and case series

Murad et al. BMJ Evid Based Med. 2018

Boyer, J.; Birzu, C.; Bielle et al. A Dramatic Response of STRN-NTRK-Fused Malignant Glioneuronal Tumor to Larotrectinib in Adult. Neuro Oncol 2021, 23, 1200–1202, doi:10.1093/neuonc/noab080.

**Domains**	**Leading explanatory questions**	**Answer**
Selection	**1.** Does the patient(s) represent(s) the whole experience of the investigator (centre) or is the selection method unclear to the extent that other patients with similar presentation may not have been reported?	0
Ascertainment	**2.** Was the exposure adequately ascertained?	1
	**3.** Was the outcome adequately ascertained?	1
Causality	**4.** Were other alternative causes that may explain the observation ruled out?	0
	**5.** Was there a challenge/rechallenge phenomenon?	0
	**6.** Was there a dose–response effect?	0
	**7.** Was follow-up long enough for outcomes to occur?	1
Reporting	**8.** Is the case(s) described with sufficient details to allow other investigators to replicate the research or to allow practitioners make inferences related to their own practice?	1

Tool for evaluating the methodological quality of case reports and case series

Murad et al. BMJ Evid Based Med. 2018

Corral Sánchez MD, Galán Gómez V, Sastre Urgelles A et al. Treatment of infantile fibrosarcoma associated to an abdominal aortic aneurysm with larotrectinib: a case report. doi: 10.1080/08880018.2021.1889730.

**Domains**	**Leading explanatory questions**	**Answer**
Selection	**1.** Does the patient(s) represent(s) the whole experience of the investigator (centre) or is the selection method unclear to the extent that other patients with similar presentation may not have been reported?	0
Ascertainment	**2.** Was the exposure adequately ascertained?	1
	**3.** Was the outcome adequately ascertained?	1
Causality	**4.** Were other alternative causes that may explain the observation ruled out?	0
	**5.** Was there a challenge/rechallenge phenomenon?	0
	**6.** Was there a dose–response effect?	0
	**7.** Was follow-up long enough for outcomes to occur?	1
Reporting	**8.** Is the case(s) described with sufficient details to allow other investigators to replicate the research or to allow practitioners make inferences related to their own practice?	1

**Tool for evaluating the methodological quality of case reports and case series**
Murad et al. BMJ Evid Based Med. 2018
Goh, X.N.; Seng, M.S.-F.; Loh, A.H.P et al.Larotrectinib Followed by Selitrectinib in a Novel DCTN1-NTRK1 Fusion Undifferentiated Pleomorphic Sarcoma. J Oncol Pharm Pract 2021, 27, 485–489, doi:10.1177/1078155220938849.

Domains	Leading explanatory questions	Answer
Selection	**1.** Does the patient(s) represent(s) the whole experience of the investigator (centre) or is the selection method unclear to the extent that other patients with similar presentation may not have been reported?	1
Ascertainment	**2.** Was the exposure adequately ascertained?	1
	**3.** Was the outcome adequately ascertained?	0
Causality	**4.** Were other alternative causes that may explain the observation ruled out?	1
	**5.** Was there a challenge/rechallenge phenomenon?	0
	**6.** Was there a dose–response effect?	0
	**7.** Was follow-up long enough for outcomes to occur?	1
Reporting	**8.** Is the case(s) described with sufficient details to allow other investigators to replicate the research or to allow practitioners make inferences related to their own practice?	1

Tool for evaluating the methodological quality of case reports and case series

Murad et al. BMJ Evid Based Med. 2018

Carter-Febres M, Schneller N, Fair D, Solomon D et al. Adjuvant Maintenance Larotrectinib Therapy in 2 Children With NTRK Fusion-positive High-grade Cancers. J Pediatr Hematol Oncol. 2021 Oct 1;43(7):e987-e990. doi: 10.1097/MPH.0000000000001983.

Domains	Leading explanatory questions	Answer
Selection	**1.** Does the patient(s) represent(s) the whole experience of the investigator (centre) or is the selection method unclear to the extent that other patients with similar presentation may not have been reported?	1
Ascertainment	**2.** Was the exposure adequately ascertained?	1
	**3.** Was the outcome adequately ascertained?	1
Causality	**4.** Were other alternative causes that may explain the observation ruled out?	0
	**5.** Was there a challenge/rechallenge phenomenon?	0
	**6.** Was there a dose–response effect?	0
	**7.** Was follow-up long enough for outcomes to occur?	1
Reporting	**8.** Is the case(s) described with sufficient details to allow other investigators to replicate the research or to allow practitioners make inferences related to their own practice?	1

Tool for evaluating the methodological quality of case reports and case series

Murad et al. BMJ Evid Based Med. 2018

Slomovic A, Amaral T, Lobko I, Siegel DN et al. Comment on: A newborn with a large NTRK fusion positive infantile fibrosarcoma successfully treated with larotrectinib. Pediatr Blood Cancer. 2021 Jun;68(6):e28953. doi: 10.1002/pbc.28953.

Domains	Leading explanatory questions	Answer
Selection	**1.** Does the patient(s) represent(s) the whole experience of the investigator (centre) or is the selection method unclear to the extent that other patients with similar presentation may not have been reported?	1
Ascertainment	**2.** Was the exposure adequately ascertained?	1
	**3.** Was the outcome adequately ascertained?	1
Causality	**4.** Were other alternative causes that may explain the observation ruled out?	0
	**5.** Was there a challenge/rechallenge phenomenon?	0
	**6.** Was there a dose–response effect?	0
	**7.** Was follow-up long enough for outcomes to occur?	1
Reporting	**8.** Is the case(s) described with sufficient details to allow other investigators to replicate the research or to allow practitioners make inferences related to their own practice?	1

Tool for evaluating the methodological quality of case reports and case series
Murad et al. BMJ Evid Based Med. 2018
Waters TW, Moore SA, Sato Y, et all. Refractory infantile high-grade glioma containing TRK-fusion responds to larotrectinib. Pediatr Blood Cancer. 2021 May;68(5):e28868. doi: 10.1002/pbc.28868.

Domains	Leading explanatory questions	Answer
Selection	**1.** Does the patient(s) represent(s) the whole experience of the investigator (centre) or is the selection method unclear to the extent that other patients with similar presentation may not have been reported?	1
Ascertainment	**2.** Was the exposure adequately ascertained?	1
	**3.** Was the outcome adequately ascertained?	1
Causality	**4.** Were other alternative causes that may explain the observation ruled out?	1
	**5.** Was there a challenge/rechallenge phenomenon?	0
	**6.** Was there a dose–response effect?	1
	**7.** Was follow-up long enough for outcomes to occur?	1
Reporting	**8.** Is the case(s) described with sufficient details to allow other investigators to replicate the research or to allow practitioners make inferences related to their own practice?	0

Tool for evaluating the methodological quality of case reports and case series

Murad et al. BMJ Evid Based Med. 2018

Mangum R, Reuther J, Bertrand KC, et all. Durable Response to Larotrectinib in a Child With Histologic Diagnosis of Recurrent Disseminated Ependymoma Discovered to Harbor an NTRK2 Fusion: The Impact of Integrated Genomic Profiling. JCO Precis Oncol. 2021 Jul 28;5:PO.20.00375. doi: 10.1200/PO.20.00375.

Domains	Leading explanatory questions	Answer
Selection	**1.** Does the patient(s) represent(s) the whole experience of the investigator (centre) or is the selection method unclear to the extent that other patients with similar presentation may not have been reported?	1
Ascertainment	**2.** Was the exposure adequately ascertained?	1
	**3.** Was the outcome adequately ascertained?	1
Causality	**4.** Were other alternative causes that may explain the observation ruled out?	1
	**5.** Was there a challenge/rechallenge phenomenon?	0
	**6.** Was there a dose–response effect?	0
	**7.** Was follow-up long enough for outcomes to occur?	0
Reporting	**8.** Is the case(s) described with sufficient details to allow other investigators to replicate the research or to allow practitioners make inferences related to their own practice?	1

Tool for evaluating the methodological quality of case reports and case series

Murad et al. BMJ Evid Based Med. 2018

Endo Y, Watanabe T, Saito M et al. A rare case of recurrent ovarian cancer with TPM3-NTRK1 gene rearrangement: A case report. Mol Clin Oncol. 2022 Apr;16(4):90. doi: 10.3892/mco.2022.2523.

Domains	Leading explanatory questions	Answer
Selection	**1.** Does the patient(s) represent(s) the whole experience of the investigator (centre) or is the selection method unclear to the extent that other patients with similar presentation may not have been reported?	1
Ascertainment	**2.** Was the exposure adequately ascertained?	1
	**3.** Was the outcome adequately ascertained?	1
Causality	**4.** Were other alternative causes that may explain the observation ruled out?	0
	**5.** Was there a challenge/rechallenge phenomenon?	0
	**6.** Was there a dose–response effect?	1
	**7.** Was follow-up long enough for outcomes to occur?	1
Reporting	**8.** Is the case(s) described with sufficient details to allow other investigators to replicate the research or to allow practitioners make inferences related to their own practice?	1

Tool for evaluating the methodological quality of case reports and case series

Murad et al. BMJ Evid Based Med. 2018

Ernst MS, Lysack JT, Hyrcza MD et al. TRK Inhibition with Entrectinib in Metastatic Salivary Secretory Carcinoma (SC): A Case Report. Curr Oncol. 2022 May 31;29(6):3933-3939. doi: 10.3390/curroncol29060314.

Domains	Leading explanatory questions	Answer
Selection	**1.** Does the patient(s) represent(s) the whole experience of the investigator (centre) or is the selection method unclear to the extent that other patients with similar presentation may not have been reported?	1
Ascertainment	**2.** Was the exposure adequately ascertained?	1
	**3.** Was the outcome adequately ascertained?	1
Causality	**4.** Were other alternative causes that may explain the observation ruled out?	0
	**5.** Was there a challenge/rechallenge phenomenon?	0
	**6.** Was there a dose–response effect?	1
	**7.** Was follow-up long enough for outcomes to occur?	1
Reporting	**8.** Is the case(s) described with sufficient details to allow other investigators to replicate the research or to allow practitioners make inferences related to their own practice?	1

Tool for evaluating the methodological quality of case reports and case series

Murad et al. BMJ Evid Based Med. 2018

Recine F, De Vita A, Fausti V et al. Case Report: Adult NTRK-Rearranged Spindle Cell Neoplasm: Early Tumor Shrinkage in a Case With Bone and Visceral Metastases Treated With Targeted Therapy. Front Oncol. 2022 Jan 7;11:740676. doi: 10.3389/fonc.2021.740676.

Domains	Leading explanatory questions	Answer
Selection	**1.** Does the patient(s) represent(s) the whole experience of the investigator (centre) or is the selection method unclear to the extent that other patients with similar presentation may not have been reported?	1
Ascertainment	**2.** Was the exposure adequately ascertained?	1
	**3.** Was the outcome adequately ascertained?	1
Causality	**4.** Were other alternative causes that may explain the observation ruled out?	0
	**5.** Was there a challenge/rechallenge phenomenon?	0
	**6.** Was there a dose–response effect?	1
	**7.** Was follow-up long enough for outcomes to occur?	1
Reporting	**8.** Is the case(s) described with sufficient details to allow other investigators to replicate the research or to allow practitioners make inferences related to their own practice?	1

Tool for evaluating the methodological quality of case reports and case series

Murad et al. BMJ Evid Based Med. 2018

Bill R, Deschler DG, Pittet MJ et al. Diagnostic challenges and successful organ-preserving therapy in a case of secretory carcinoma of minor salivary glands. doi: 10.1002/cnr2.1491.

Domains	Leading explanatory questions	Answer
Selection	**1.** Does the patient(s) represent(s) the whole experience of the investigator (centre) or is the selection method unclear to the extent that other patients with similar presentation may not have been reported?	0
Ascertainment	**2.** Was the exposure adequately ascertained?	1
	**3.** Was the outcome adequately ascertained?	1
Causality	**4.** Were other alternative causes that may explain the observation ruled out?	1
	**5.** Was there a challenge/rechallenge phenomenon?	0
	**6.** Was there a dose–response effect?	0
	**7.** Was follow-up long enough for outcomes to occur?	1
Reporting	**8.** Is the case(s) described with sufficient details to allow other investigators to replicate the research or to allow practitioners make inferences related to their own practice?	1

Tool for evaluating the methodological quality of case reports and case series

Murad et al. BMJ Evid Based Med. 2018

Bargas, S.; Mc Leer, A.; Mondet, et al. An Impressive Response with Larotrectinib in a Patient with a Papillary Thyroid Carcinoma Harboring an SQSTM1-NTRK1 Fusion. doi:10.1530/EJE-21-0509.

Domains	Leading explanatory questions	Answer
Selection	**1.** Does the patient(s) represent(s) the whole experience of the investigator (centre) or is the selection method unclear to the extent that other patients with similar presentation may not have been reported?	0
Ascertainment	**2.** Was the exposure adequately ascertained?	1
	**3.** Was the outcome adequately ascertained?	1
Causality	**4.** Were other alternative causes that may explain the observation ruled out?	1
	**5.** Was there a challenge/rechallenge phenomenon?	0
	**6.** Was there a dose–response effect?	0
	**7.** Was follow-up long enough for outcomes to occur?	1
Reporting	**8.** Is the case(s) described with sufficient details to allow other investigators to replicate the research or to allow practitioners make inferences related to their own practice?	1

Tool for evaluating the methodological quality of case reports and case series

Murad et al. BMJ Evid Based Med. 2018

Kasi, P.M.; Afghan, M.K.; Bellizzi, A.M.; et al. Larotrectinib in Mismatch-Repair-Deficient TRK Fusion-Positive Metastatic Colon Cancer After Progression on Immunotherapy.doi:10.7759/cureus.26648.

Domains	Leading explanatory questions	Answer
Selection	**1.** Does the patient(s) represent(s) the whole experience of the investigator (centre) or is the selection method unclear to the extent that other patients with similar presentation may not have been reported?	0
Ascertainment	**2.** Was the exposure adequately ascertained?	0
	**3.** Was the outcome adequately ascertained?	1
Causality	**4.** Were other alternative causes that may explain the observation ruled out?	0
	**5.** Was there a challenge/rechallenge phenomenon?	0
	**6.** Was there a dose–response effect?	0
	**7.** Was follow-up long enough for outcomes to occur?	1
Reporting	**8.** Is the case(s) described with sufficient details to allow other investigators to replicate the research or to allow practitioners make inferences related to their own practice?	1

Tool for evaluating the methodological quality of case reports and case series

Murad et al. BMJ Evid Based Med. 2018

Saliba M, Mohanty AS, Ho AL et al. Secretory Carcinoma of the Thyroid in a 49-Year-Old Man Treated with Larotrectinib: Protracted Clinical Course of Disease Despite the High-Grade Histologic Features. doi: 10.1007/s12105-021-01386-6.

Domains	Leading explanatory questions	Answer
Selection	**1.** Does the patient(s) represent(s) the whole experience of the investigator (centre) or is the selection method unclear to the extent that other patients with similar presentation may not have been reported?	0
Ascertainment	**2.** Was the exposure adequately ascertained?	0
	**3.** Was the outcome adequately ascertained?	0
Causality	**4.** Were other alternative causes that may explain the observation ruled out?	1
	**5.** Was there a challenge/rechallenge phenomenon?	0
	**6.** Was there a dose–response effect?	0
	**7.** Was follow-up long enough for outcomes to occur?	1
Reporting	**8.** Is the case(s) described with sufficient details to allow other investigators to replicate the research or to allow practitioners make inferences related to their own practice?	0

Tool for evaluating the methodological quality of case reports and case series

Murad et al. BMJ Evid Based Med. 2018

Lapeña LM, Caldas MCS, Ramírez C et al. Larotrectinib as an Effective Therapy in Congenital Infantile Fibrosarcoma: Report of Two Cases. doi: 10.1055/s-0042-1748866.

Domains	Leading explanatory questions	Answer
Selection	**1.** Does the patient(s) represent(s) the whole experience of the investigator (centre) or is the selection method unclear to the extent that other patients with similar presentation may not have been reported?	0
Ascertainment	**2.** Was the exposure adequately ascertained?	0
	**3.** Was the outcome adequately ascertained?	0
Causality	**4.** Were other alternative causes that may explain the observation ruled out?	1
	**5.** Was there a challenge/rechallenge phenomenon?	0
	**6.** Was there a dose–response effect?	0
	**7.** Was follow-up long enough for outcomes to occur?	1
Reporting	**8.** Is the case(s) described with sufficient details to allow other investigators to replicate the research or to allow practitioners make inferences related to their own practice?	0

Tool for evaluating the methodological quality of case reports and case series

Murad et al. BMJ Evid Based Med. 2018

Groussin L, Theodon H, Bessiene L, et al. Redifferentiating Effect of Larotrectinib in NTRK-Rearranged Advanced Radioactive-Iodine Refractory Thyroid Cancer. Thyroid. 2022 May;32(5):594-598. doi: 10.1089/thy.2021.0524.

Domains	Leading explanatory questions	Answer
Selection	**1.** Does the patient(s) represent(s) the whole experience of the investigator (centre) or is the selection method unclear to the extent that other patients with similar presentation may not have been reported?	1
Ascertainment	**2.** Was the exposure adequately ascertained?	1
	**3.** Was the outcome adequately ascertained?	1
Causality	**4.** Were other alternative causes that may explain the observation ruled out?	0
	**5.** Was there a challenge/rechallenge phenomenon?	0
	**6.** Was there a dose–response effect?	1
	**7.** Was follow-up long enough for outcomes to occur?	1
Reporting	**8.** Is the case(s) described with sufficient details to allow other investigators to replicate the research or to allow practitioners make inferences related to their own practice?	1

Tool for evaluating the methodological quality of case reports and case series

Murad et al. BMJ Evid Based Med. 2018

Grogan PT, Deming DA, Helgager J, et al. Entrectinib demonstrates prolonged efficacy in an adult case of radiation-refractory NTRK fusion glioblastoma. Neurooncol Adv. 2022 Apr 13;4(1):vdac046. doi: 10.1093/noajnl/vdac046.

Domains	Leading explanatory questions	Answer
Selection	**1.** Does the patient(s) represent(s) the whole experience of the investigator (centre) or is the selection method unclear to the extent that other patients with similar presentation may not have been reported?	1
Ascertainment	**2.** Was the exposure adequately ascertained?	0
	**3.** Was the outcome adequately ascertained?	1
Causality	**4.** Were other alternative causes that may explain the observation ruled out?	1
	**5.** Was there a challenge/rechallenge phenomenon?	0
	**6.** Was there a dose–response effect?	1
	**7.** Was follow-up long enough for outcomes to occur?	0
Reporting	**8.** Is the case(s) described with sufficient details to allow other investigators to replicate the research or to allow practitioners make inferences related to their own practice?	1

Tool for evaluating the methodological quality of case reports and case series

Murad et al. BMJ Evid Based Med. 2018

Kobayashi H, Makise N, Shinozaki-Ushiku A, et al. Dramatic response to entrectinib in a patient with malignant peripheral nerve sheath tumor harboring novel SNRNP70-NTRK3 fusion gene. Genes Chromosomes Cancer. 2022 Jul 30. doi: 10.1002/gcc.23089.

Domains	Leading explanatory questions	Answer
Selection	**1.** Does the patient(s) represent(s) the whole experience of the investigator (centre) or is the selection method unclear to the extent that other patients with similar presentation may not have been reported?	1
Ascertainment	**2.** Was the exposure adequately ascertained?	1
	**3.** Was the outcome adequately ascertained?	1
Causality	**4.** Were other alternative causes that may explain the observation ruled out?	0
	**5.** Was there a challenge/rechallenge phenomenon?	0
	**6.** Was there a dose–response effect?	0
	**7.** Was follow-up long enough for outcomes to occur?	1
Reporting	**8.** Is the case(s) described with sufficient details to allow other investigators to replicate the research or to allow practitioners make inferences related to their own practice?	1

**Tool for evaluating the methodological quality of case reports and case series**
Murad et al. BMJ Evid Based Med. 2018
König D, Hench J, Frank S, et al. Larotrectinib Response in NTRK3 Fusion-Driven Diffuse High-Grade Glioma. Pharmacology. 2022;107(7-8):433-438. doi: 10.1159/000524399.

Domains	Leading explanatory questions	Answer
Selection	**1.** Does the patient(s) represent(s) the whole experience of the investigator (centre) or is the selection method unclear to the extent that other patients with similar presentation may not have been reported?	1
Ascertainment	**2.** Was the exposure adequately ascertained?	1
	**3.** Was the outcome adequately ascertained?	1
Causality	**4.** Were other alternative causes that may explain the observation ruled out?	0
	**5.** Was there a challenge/rechallenge phenomenon?	1
	**6.** Was there a dose–response effect?	1
	**7.** Was follow-up long enough for outcomes to occur?	1
Reporting	**8.** Is the case(s) described with sufficient details to allow other investigators to replicate the research or to allow practitioners make inferences related to their own practice?	1

Tool for evaluating the methodological quality of case reports and case series

Murad et al. BMJ Evid Based Med. 2018

Olsen MR, Denu RA, Lyon JB, Gulliver JM et al. Undifferentiated and Unresectable Sarcoma With NTRK3-Fusion in a Pediatric Patient Treated With Larotrectinib and Proton Beam Radiotherapy. J Pediatr Hematol Oncol. 2022 Apr 1;44(3):e770-e774. doi: 10.1097/MPH.0000000000002358.

Domains	Leading explanatory questions	Answer
Selection	**1.** Does the patient(s) represent(s) the whole experience of the investigator (centre) or is the selection method unclear to the extent that other patients with similar presentation may not have been reported?	1
Ascertainment	**2.** Was the exposure adequately ascertained?	1
	**3.** Was the outcome adequately ascertained?	1
Causality	**4.** Were other alternative causes that may explain the observation ruled out?	0
	**5.** Was there a challenge/rechallenge phenomenon?	0
	**6.** Was there a dose–response effect?	0
	**7.** Was follow-up long enough for outcomes to occur?	1
Reporting	**8.** Is the case(s) described with sufficient details to allow other investigators to replicate the research or to allow practitioners make inferences related to their own practice?	1

Tool for evaluating the methodological quality of case reports and case series

Murad et al. BMJ Evid Based Med. 2018

Mançano BM, Dos Reis MB, Moreno DA et al. A Unique Case Report of Infant-Type Hemispheric Glioma (Gliosarcoma Subtype) with TPR-NTRK1 Fusion Treated with Larotrectinib. Pathobiology. 2022;89(3):178-185. doi: 10.1159/000521253.

Domains	Leading explanatory questions	Answer
Selection	**1.** Does the patient(s) represent(s) the whole experience of the investigator (centre) or is the selection method unclear to the extent that other patients with similar presentation may not have been reported?	1
Ascertainment	**2.** Was the exposure adequately ascertained?	1
	**3.** Was the outcome adequately ascertained?	1
Causality	**4.** Were other alternative causes that may explain the observation ruled out?	0
	**5.** Was there a challenge/rechallenge phenomenon?	0
	**6.** Was there a dose–response effect?	0
	**7.** Was follow-up long enough for outcomes to occur?	0
Reporting	**8.** Is the case(s) described with sufficient details to allow other investigators to replicate the research or to allow practitioners make inferences related to their own practice?	1

Tool for evaluating the methodological quality of case reports and case series

Murad et al. BMJ Evid Based Med. 2018

Di Ruscio V, Carai A, Del Baldo G, Vinci M, Cacchione A, Miele E, Rossi S, Antonelli M, Barresi S, Caulo M, Colafati GS, Mastronuzzi A. Molecular Landscape in Infant High-Grade Gliomas: A Single Center Experience. Diagnostics (Basel). 2022 Feb 1;12(2):372. doi: 10.3390/diagnostics12020372.

Domains	Leading explanatory questions	Answer
Selection	**1.** Does the patient(s) represent(s) the whole experience of the investigator (centre) or is the selection method unclear to the extent that other patients with similar presentation may not have been reported?	1
Ascertainment	**2.** Was the exposure adequately ascertained?	1
	**3.** Was the outcome adequately ascertained?	1
Causality	**4.** Were other alternative causes that may explain the observation ruled out?	0
	**5.** Was there a challenge/rechallenge phenomenon?	0
	**6.** Was there a dose–response effect?	0
	**7.** Was follow-up long enough for outcomes to occur?	1
Reporting	**8.** Is the case(s) described with sufficient details to allow other investigators to replicate the research or to allow practitioners make inferences related to their own practice?	1
